# Recombination Modulates How Selection Affects Linked Sites in *Drosophila*

**DOI:** 10.1371/journal.pbio.1001422

**Published:** 2012-11-13

**Authors:** Suzanne E. McGaugh, Caiti S. S. Heil, Brenda Manzano-Winkler, Laurence Loewe, Steve Goldstein, Tiffany L. Himmel, Mohamed A. F. Noor

**Affiliations:** 1 Biology Department, Duke University, Durham, North Carolina, United States of America; 2 Laboratory of Genetics and Wisconsin Institute for Discovery, University of Wisconsin–Madison, Madison, Wisconsin, United States of America; 3 Duke Institute for Genome Sciences and Policy, Duke University, Durham, North Carolina, United States of America; Institute of Science and Technology Austria (IST Austria), Austria

## Abstract

One of the most influential observations in molecular evolution has been a strong association between local recombination rate and nucleotide polymorphisms across the genome. This is interpreted as evidence for ubiquitous natural selection. The alternative explanation, that recombination is mutagenic, has been rejected by the absence of a similar association between local recombination rate and nucleotide divergence between species. However, many recent studies show that recombination rates are often very different even in closely related species, questioning whether an association between recombination rate and divergence between species has been tested satisfactorily. To circumvent this problem, we directly surveyed recombination across approximately 43% of the *D. pseudoobscura* physical genome in two separate recombination maps and 31% of the *D. miranda* physical genome, and we identified both global and local differences in recombination rate between these two closely related species. Using only regions with conserved recombination rates between and within species and accounting for multiple covariates, our data support the conclusion that recombination is positively related to diversity because recombination modulates Hill–Robertson effects in the genome and not because recombination is predominately mutagenic. Finally, we find evidence for dips in diversity around nonsynonymous substitutions. We infer that at least some of this reduction in diversity resulted from selective sweeps and examine these dips in the context of recombination rate.

## Abstract 2

Recombination rate in *Drosophila* species shapes the impact of selection in the genome and is positively correlated with nucleotide diversity.

## Author Summary

Individuals within a species differ in the DNA sequences of their genes. This sequence variation affects how well individuals survive or reproduce and is transmitted to their offspring. Genes near each other on individual chromosomes tend to be passed to offspring together—neighboring genes are unlikely to be separated by exchanges of genetic material derived from different parents during meiotic recombination. When genes are inherited together, however, the evolutionary forces acting on one gene can interfere with variation at its neighbors. Thus, variation at multiple genes can be lost if natural selection acts on one gene in close proximity. Recombination can prevent or reduce this loss of variation, but previous tests of this phenomenon failed to account for recombination rate differences between species. In this study, we show that some parts of the genome differ in recombination rate between two species of fruit fly, *Drosophila pseudoobscura* and *D. miranda*. Avoiding an assumption made in previous studies, we then examine sequence variation within and between fly species in those parts of the genome that have conserved recombination rates. Based on the results, we conclude that recombination indeed preserves variation within species that would otherwise have been eliminated by natural selection.

## Introduction

Homologous meiotic recombination has an important role in molecular evolution. Sufficient recombination uncouples the evolution of different sites on the same chromosome allowing positive or negative selection at one site to act independently from selection at another site. If there is less than effectively free recombination between two selected sites, then linkage results in selection at one site interfering with selection at another site. This has been termed “Hill–Roberson interference” [1]–[6]. Hill–Robertson interference increases the probability of fixation of deleterious mutations, decreases the probability of fixation of advantageous mutations, and reduces overall DNA sequence diversity. Thus, the breakdown of linkage disequilibrium between loci experiencing Hill–Robertson interference allows selection to act more efficiently, purging deleterious mutations and accelerating adaptation [1]–[6].

Such indirect effects of recombination on the genome [7] result in a positive association between the rate of recombination and adaptive evolution [8]–[10]. For example, recombination rate is positively associated with codon usage bias, whereby those codons coded by the most abundant tRNAs are “preferred” and used more often [11],[12]. Recombination has direct effects on a genome sequence as well, because recombination influences base composition through biased gene conversion and the distribution of repetitive elements, hotspot sequences, and indels [7],[13]–[17]. Understanding the magnitude of indirect effects in light of these direct effects has proved challenging [7].

One striking association is a positive relationship of local recombination rate and nucleotide diversity [13],[18],[19]. Originally described in *Drosophila melanogaster* [13], the positive relationship between recombination rate and nucleotide diversity has been demonstrated in a wide range of taxa, including humans, mice, yeast, maize, and tomatoes (reviewed in [20]). It is not fully understood how much of this relationship results from recombination's indirect versus direct effects on the genome. For instance, mutations created during crossing over or double-strand break repair may generate new polymorphisms and hence increase diversity [21]–[27]. Alternatively, recombination may indirectly influence genetic diversity by mitigating the genomic footprint of selective sweeps and background selection [28]–[30].

One way to distinguish between these general explanations is to evaluate the relationship of between-species nucleotide divergence at neutral sites and local recombination rate, because truly neutral mutations are substituted at the same average rate between species as they appear between generations, even if linked to sites under selection [31],[32]. This allows us to predict that both within-species nucleotide diversity and between-species nucleotide divergence would have a positive relationship with local recombination rate [13], if the recombination–diversity association was purely caused by mutation. In contrast, selective sweeps and background selection will cause an association between recombination and within-species nucleotide diversity, but not a relationship between recombination and between-species nucleotide divergence [30],[32]. The absence of an association of between-species nucleotide divergence and local recombination rate suggests that variation in recombination rate translates to variation in the efficiency of selection [13]. Past work relating nucleotide divergence to recombination rate found no relationship between these two variables in several species of *Drosophila*, mouse, beet, yeast, and other species [13],[20],[33]–[37]. Furthermore, in several species, evidence indicates that segregating ancestral polymorphisms may be responsible for correlations between divergence and recombination rate ([38]–[40], also suggested by [25],[41]).

The test above, however, implicitly assumes that local recombination rates are conserved between the two species used to generate the nucleotide divergence measure. If recombination rate has diverged between the two species, no relationship between local recombination rate and nucleotide divergence may be detected even when recombination is mutagenic (see Figure S1). Recombination rates, especially at fine scales, are often not conserved among closely related species, as is the case between humans and chimpanzees [42]–[44]; thus, the assumption of conservation of recombination rates may be violated in previous studies, and a more definitive understanding of the diversity–recombination association awaits estimates that are free from this assumption.

Though there are theoretical expectations concerning how recombination rate should affect selection efficiency [45],[46], it is unclear empirically whether variation in local recombination rates translates into significant variation in the efficiency of selection [7]. Several empirical studies have tackled this problem [12],[38],[47]–[52], and many findings suggest that recombination rate influences the efficiency of positive or negative selection in regions of moderate or high recombination. Still, various confounding factors (e.g., biased gene conversion, gene density) may produce spurious correlations between both recombination and substitution rate, and some authors suggest that there is no strong empirical evidence for recombination affecting the efficiency of selection (apart from reduced selection in regions with essentially no recombination [7]).

The *Drosophila pseudoobscura* system is ideal for pursuing questions about recombination rate variation and its molecular evolutionary consequences. The average crossover rate of *D. pseudoobscura* (about 7 cM/Mb in females) is over twice that of *D. melanogaster* [53]. There is also considerable fine-scale (<200 kb windows) variation in the local recombination rate within the genome of *D. pseudoobscura* and within the genome of its sister species, *D. persimilis* [25],[33],[54]. While some recombination data are available for *D. pseudoobscura* and *D. persimilis*, these sister taxa interbreed in the wild [55]–[57] and are, therefore, not ideal for examining the divergence–recombination association. For example, shared polymorphism due to hybridization and recent speciation may be responsible for the positive divergence–recombination association found in a previous study [25] (see also [38],[39]). Fortunately, a third species exists (*D. miranda*) that is phylogenetically close to *D. pseudoobscura* but does not interbreed with *D. pseudoobscura*. Since there is still some residual shared ancestral polymorphism [58], we also obtained the genome sequence for a slightly more distantly related outgroup species, *D. lowei* (Figure S2). Sequence from *D. lowei* is useful for generating a proxy for neutral mutation rate across the genome.

In this work, we generate and compare two fine-scale recombination maps for *D. pseudoobscura*, which each cover approximately 43% of the *D. pseudoobscura* physical genome and one fine-scale recombination map that covers approximately 31% of the *D. miranda* physical genome. In order to circumvent the assumption of classic studies, we analyze the relationship of local recombination rate to nucleotide diversity and divergence in regions with very similar recombination rates between the two species. By employing a linear model framework to account for multiple covariates, we conclude that the contribution of recombination to diversity is significant and positive, but recombination contributes little to divergence. This indicates that recombination is likely to modulate the footprint of selection in the genome. Next, we tested the impact of recombination rate on the efficiency of selection. We examined whether recombination rate (1) affects the distribution of nonsynonymous substitutions across the genome and (2) affects the pattern of diversity around nonsynonymous and synonymous substitutions. In particular, we use a generalized linear model to test how recombination modulates the magnitude and physical extent of the loss of diversity surrounding substitutions. Our analysis of these putative selective sweeps should be less sensitive to common confounding factors such as gene expression and GC content than previous measures. In total, this work allowed us to determine that recombination rate has an important impact on how selection shapes diversity across the genome of *Drosophila pseudoobscura* and its close relatives.

## Results

We first discuss general features of the recombination landscapes we observed in *Drosophila pseudoobscura* and *D. miranda* before we address the implications of these observations for understanding diversity, divergence, and the nature of selection in the genomes we sequenced.

### General Summary of Recombination Data: Fine-Scale Maps

We generated linkage maps for chromosome 2 and parts of the X chromosome for *D. pseudoobscura* and *D. miranda*. Using a backcross design and inbred lines, we developed two replicate recombination maps (referred to here as “Flagstaff” and “Pikes Peak”) for *D. pseudoobscura* and one recombination map for *D. miranda* using the Illumina BeadArray platform to distinguish heterozygotes from homozygotes of the inbred lines used in the backcross design. These maps (Table S1) measure recombination rate across <200 kb windows, and we refer to these as “fine-scale” maps.

Recombination was surveyed across approximately 43% of the *D. pseudoobscura* physical genome and about 31% of the *D. miranda* physical genome (Tables S1 and S2). For each of the three maps, nearly the entire assembled region of chromosome 2 (97.8%–99.4%), the majority of the XR chromosome arm (70.8%–89.4%), and part of the XL chromosome arm (∼22%–23%) were surveyed (Table S2). After removal of likely erroneous putative double recombinants, ambiguous genotypes, and markers that did not work or gave inconsistent genotypes, recombination was measured for three different crosses for 1,158–1,404 individuals per map (Table S1). Excluding larger intervals at the telomeres and centromeres, intervals between markers had a median size across the three maps of 141–148 kb for chromosome 2 and 146–160 kb for the XR chromosome arm (Table S1).

For chromosome 2, recombination rates ranged from 0–30.8 cM/Mb in *D. pseudoobscura* and 0–24.0 cM/Mb in *D. miranda* (Table S2). The number of individuals surveyed is often slightly different per interval; therefore, for all intervals where no recombination was detected, we report 0 cM/Mb. The recombination rate for those intervals with “0 cM” should be interpreted as <1 recombination event per total number of individuals surveyed for each interval (Dataset S1). Recombination near the telomere and centromere was measured at a broader scale than the remainder of chromosome 2 because we expected these regions to have lower crossover rates than the center of the chromosome (chromosome 2 is telocentric). Because of this limitation, comparisons of recombination rates between the ends of the chromosome and the center are more tentative. Nonetheless, examining recombination across roughly 3 Mb of sequence at the telomeric end and 3 Mb at the centromeric end, we found up to an 8.9-fold difference between the recombination rates at the middle of chromosome 2 relative to the centromeric end. The Pikes Peak *D. pseudoobscura* map exhibited the largest reduction of recombination at the telomeric or centromeric ends relative to the center of the chromosome for all three maps, though in the Flagstaff *D. pseudoobscura* map and the *D. miranda* map, recombination rates were reduced by at least 2.6-fold in the centromere and telomere relative to the center of the chromosome (Table S3).

For the XR chromosome arm, recombination rates ranged from 0–25.2 cM/Mb in *D. pseudoobscura* and 0–32.3 cM/Mb in *D. miranda* (Figure S3 presented with 95% confidence intervals; see also Dataset S1, Table S2). The number of crossovers per individual for both chromosome 2 and the XR arm was close to 1 (1.01–1.06) for *D. pseudoobscura* and was 1.40–1.54 for *D. miranda*, illustrating that a greater overall recombination rate in *D. miranda* relative to *D. pseudoobscura* is observed in both an autosome and a sex chromosome.

The XL chromosome arm was not surveyed as intensively (∼22%–23% of the XL arm in Pikes Peak and *D. miranda* and ∼60% of the XL arm in Flagstaff; Figure S4 presented with 95% confidence intervals; Dataset S1). The number of crossovers per individual appears consistent with ∼1 crossover per chromosome arm, as in *D. pseudoobscura* XR and chromosome 2, but the average number of crossovers per individual on the XL reflects how much of the arm was surveyed. For example, when ∼22%–23% of the arm was surveyed, crossovers per individual ranged from 0.23–0.26 (Table S2).

**Figure 1:**
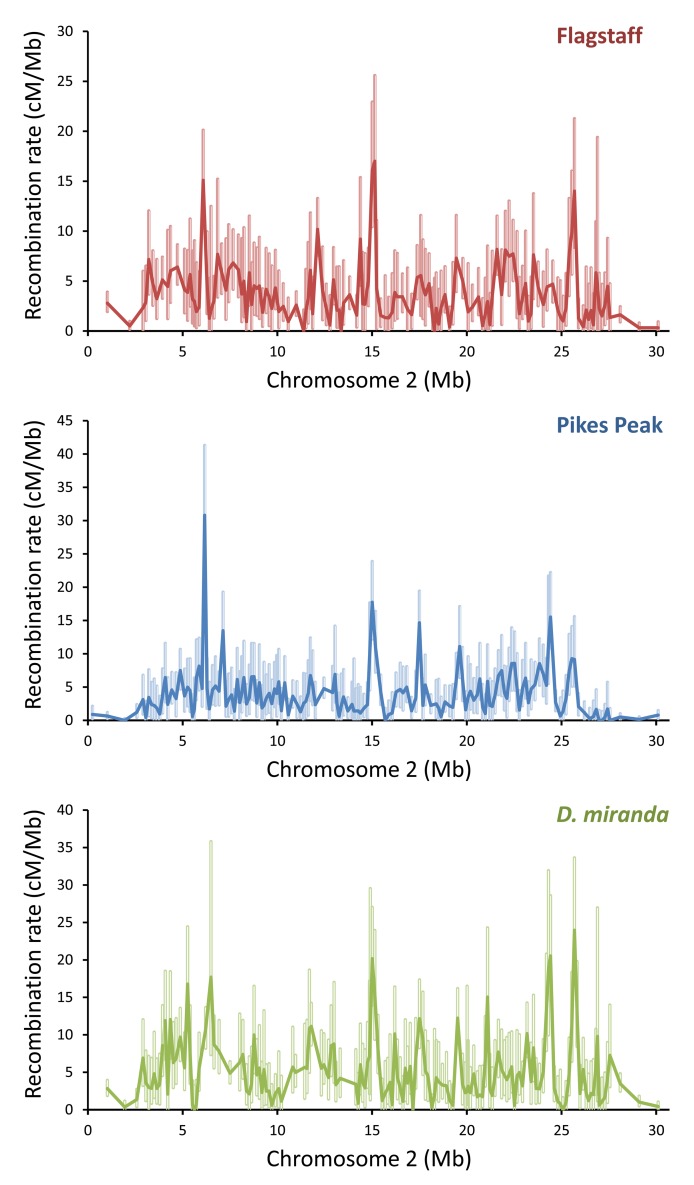
Fine-scale recombination rates on chromosome 2. Uncondensed raw recombination rates and 95% CI for intervals along chromosome 2. Top, *D. pseudoobscura* Flagstaff map; middle, *D. pseudoobscura* Pikes Peak map; bottom, *D. miranda*. Recombination rates are given in Kosambi centiMorgans per Megabase (cM/Mb).

A binomial Generalized Linear Model (GLM) with size of the interval as a covariate and interval identity as a factor in the model indicated significant heterogeneity in recombination rate among intervals for chromosome 2, XR, and XL (each tested separately) for each of the three maps (each tested separately, interval identity *p*<0.00001, χ^2^≥64.67, *df*≥3, in all cases). Furthermore, 95% confidence intervals (generated via the same method in [54]) do not overlap in many cases between different intervals (shown in Figures 1, S3, S4; Dataset S1). Overall, we observe heterogeneity in fine-scale recombination rates within each of the three maps (see Figures 1, S3, and S4 with 95% confidence intervals plotted; Dataset S1; statistical quantification between maps given in section below), and we note a reduction in recombination rate around the telomeric and centromeric ends consistent with other studies in *Drosophila* [33].

### General Summary of Recombination Data: Ultrafine-Scale Maps

**Figure 2:**
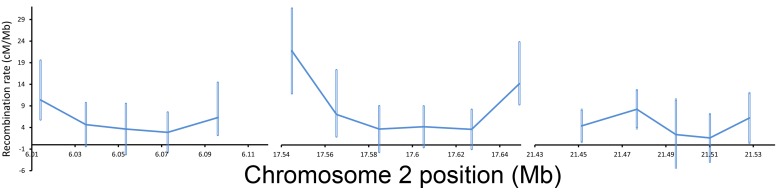
Ultrafine recombination rates. Recombination rates in Kosambi cM/Mb and 95% CI for ultrafine intervals along chromosome 2.

Our three fine-scale crossover maps utilized markers on average 141–160 kb apart (median interval size for each of the three maps, with the exception of XL where the median distance between markers was 200–1,775 kb for the three crosses). We additionally examined three regions on chromosome 2 in more detail. Each of these regions spanned a total of 99–125 kb, and we placed markers every ∼20 kb within the region (16 total intervals; Tables S4 and S5). These regions were originally picked because previous data [25],[33] indicated that recombination rates for each of these regions differed (regions are referred to as 6 Mb, 17 Mb, and 21 Mb, which indicate approximate location on chromosome 2). We refer to these as “ultrafine-scale” maps. For these ultrafine maps, we followed the same backcross scheme as above, and we scored approximately 10,000 individuals for each marker (Table S5). For the 16 ultrafine intervals (Tables S4 and S5), each interval was on average 20.61 kb long (range 12.6–27.4 kb). Recombination rates range from 1.6–21.2 cM/Mb for these ∼20 kb intervals (Figure 2; see Table S5 for 95% CI). The ultrafine-scale map uncovered variation in recombination rates that was not apparent with the fine-scale maps. For example, for the 17 Mb ultrafine-scale region on chromosome 2, the recombination rates for the two fine-scale intervals spanning this region (17.5–17.7 Mb) are 5.6 and 4.4 cM/Mb. The ultrafine-scale recombination rates, in contrast, ranged from 3.5–21.2 cM/Mb (markers spanning 17.5–17.7 Mb). This heterogeneity in recombination rates within the ultrafine regions was statistically significant (binomial GLM similar to that described in fine-scale section above: *p* = 0.0011, *df* = 14, χ^2^ = 35.91; 95% confidence intervals given in Table S5) and highlights the fact that “broader” scale measures of recombination rates (such as the fine-scale measures here) are averages of true variation in recombination rate.

### Recombination Rate Comparison between Maps

For comparisons of recombination rates between fine-scale maps, we restricted our analysis to intervals that were condensed to have nearly identical physical marker placement between the three fine-scale maps (Figures S5 and S6; Table S6). Recombination was estimated as detailed above, using the number of crossovers spanning the newly defined physical intervals. After condensing across all three maps, 97 intervals remained for chromosome 2 and 44 intervals for XR (see Tables 1 and S6 fornumber of individuals, size, range of these condensed intervals,and base pairs between markers on each map). The XL chromosome arm was not included in the analysis that used condensed intervals across maps because too few intervals overlapped between all three maps. When comparing two maps, intervals were condensed between those two maps only (see Datasets S2 and S3 for rare events logistic regressions for all two-map and three-map comparisons).

**Table 1:** Comparison of intervals condensed within and between recombination maps.

Map Comparisons	Parameter	Ch2 (*N* = 97)	XR (*N* = 44)
*D. pseudoobscura* Pikes Peak–Flagstaff	Different/conserved	1/60	0/21
	Odds Ratio	0.9789 (0.8682, 1.1037)	1.0602 (0.8700, 1.2919)
	*p* value	*p*<0.727	*p*<0.562
*D. pseudoobscura* Flagstaff–*D. miranda*	Different/conserved	0/50	2/20
	Odds Ratio	0.7794 (0.6916, 0.8787)	0.5860 (0.4877, 0.7041)
	*p*-value	*p*<0.001*	*p*<0.001*
*D. pseudoobscura* Pikes Peak–*D. miranda*	Different/conserved	3/48	5/19
	Odds Ratio	0.7629 (0.6780, 0.8584)	0.6213 (0.5267, 0.7328)
	*p* value	*p*<0.001*	*p*<0.001*

The number of significantly different and conserved intervals between each set of maps is given (criteria outlined in text). In defining significantly different intervals, we performed a false discovery rate correction of [59]. The Odds Ratio and associated *p* value are given for the difference between maps for the condensed intervals.

* *p*<0.05.

### Local Recombination Rates between Two *D. pseudoobscura* Maps Are Similar

Recombination rates did not differ significantly between the two *D. pseudoobscura* maps for either the XR or chromosome 2 for the two-map comparisons (each chromosome analyzed separately, rare events logistic regression, absolute value of *z*>0.3901, *p*>0.236, in both cases; Dataset S2). For chromosome 2, one interval was significantly different in recombination rate after correcting for multiple tests [59]. For the XR, no intervals between the two *D. pseudoobscura* maps were significantly different in recombination rate after correcting for multiple tests. The 95% confidence intervals for the odds ratio of the difference between maps were narrow and located around zero, indicating that the maps are likely very similar (chromosome 2, 0.87–1.10; XR, 0.94, 1.28; within-species two map comparison). It is unlikely that the single significant difference observed within the same species is because of slight differences in marker placement between the two maps. The marker placement for this interval was nearly identical between the two maps (left marker, 102 nucleotides different between maps; right marker, 17 nucleotides).

### Globally Higher Recombination Rate in *D. miranda* Relative to *D. pseudoobscura*

For both chromosome 2 and the XR chromosome arm, *Drosophila miranda* had significantly higher recombination rates than both *D. pseudoobscura* maps (Figure S5, Table 1, Datasets S2 and S3). A rare events logistic regression of two-map comparisons indicated that the recombination rate of the *D. pseudoobscura* crosses we surveyed is about 76%–78% of the *D. miranda* recombination rate we observed on chromosome 2 (absolute *z* value>4.5374, *p*<0.001 for *D. miranda* relative to either *D. pseudoobscura* map, Table 1). The recombination rate of *D. pseudoobscura* is about 68%–71% of the *D. miranda* recombination rate on the XR chromosome arm (rare events logistic regression absolute *z* value>5.101, *p*<0.001 for *D. miranda* relative to either *D. pseudoobscura* map, Table 1).

### Limited Local Recombination Rate Divergence between *D. pseudoobscura* and *D. miranda*

After the global difference between *D. miranda* and *D. pseudoobscura* is accounted for by the rare events logistic regression, recombination rates within and between species appear very similar for chromosome 2 (Figure S5; Datasets S2 and S3). None of the intervals for the two-map comparison between *D. miranda* and *D. pseudoobscura*–Flagstaff were significantly different after correction for multiple tests, though power to detect significant differences on a per interval basis was likely weak (see confidence intervals in Datasets S2 and S3). For example, 15 of the 115 intervals on chromosome 2 showed at least a 3-fold difference in recombination rate between maps (Datasets S2 and S3), though this magnitude of difference was not significant in our rare events logistic regression after correcting for multiple tests. Likewise, only one of the intervals for the two-map comparison between *D. miranda* and *D. pseudoobscura*–Pikes Peak was significantly different after correction for multiple tests, but 19 of the 123 intervals exhibited at least a 3-fold difference in recombination rate between maps for chromosome 2.

The XR chromosome exhibited a qualitatively larger difference in recombination rate between species than chromosome 2. After the global difference between *D. miranda* and *D. pseudoobscura* is accounted for by a rare events logistic regression, two of the intervals between *D. miranda* and *D. pseudoobscura*–Flagstaff for the two-map comparison and seven of the intervals between the *D. miranda* and *D. pseudoobscura*–Pikes Peak two-map comparison were significantly different after correction for multiple tests. Six of the 72 intervals between *D. miranda* and *D. pseudoobscura*–Flagstaff two-map comparison exhibited at least a 3-fold difference, and 12 of 102 intervals between *D. miranda* and *D. pseudoobscura*–Pikes Peak exhibited at least a 3-fold difference (Dataset S2).

Twenty-seven of 97 condensed intervals (three-map comparison, condensed between all three maps) for chromosome 2 were considered to be “conserved” within and between species. This means that they displayed a nonsignificant difference across all three maps when analyzed with a rare events logistic regression and had an odds ratio between 0.62 and 1.615 after the effect of map identity was taken into account. These “conserved” intervals were used for further downstream analyses (see “Diversity, Divergence, and Recombination”; Table S7). For the XR, seven of 44 intervals condensed between all three maps were conserved within and between species according to the criteria outlined above.

In sum, we observe strong conservation in recombination rates within a single species, while between species, we see globally elevated recombination rates in *D. miranda*. Once the global difference is accounted for, there are few intervals with significant differences in recombination rate within and between species. Thus, it is possible and parsimonious that recombination rate is generally conserved at the scale examined here (∼180 kb) over moderate evolutionary timescales (2–2.5 my).

### Diversity, Divergence, and Recombination

We used various Illumina platforms to resequence genomic DNA from 10 *D. pseudoobscura* lines using virgin females from lines that were inbred for five or more generations with full-sibling single-pair mating (Table S8). *Drosophila pseudoobscura* populations across North America display very little differentiation, as indicated by low F_ST_ values (always<0.10, often<0.05 for loci located outside of the inversion polymorphisms of the third chromosome) [60],[61]. Therefore, the choice of strains sequenced for estimating diversity covered much of the species range but was fairly random. We also sequenced two lines of *D. persimilis* (one of these was provided by S. Nuzhdin), two lines of *D. pseudoobscura bogotana* (one of these was provided by S. Nuzhdin), one line of *D. lowei*, and three lines of *D. miranda* (two provided by D. Bachtrog, Table S8; Short Read Archive accession numbers SRA044960.1, SRA044955.2, and SRA044956.1; see also http://pseudobase.biology.duke.edu/). The divergence between *D. persimilis* and *D. lowei* was used to generate measures of a proxy for neutral mutation rate across the genome. In all diversity and divergence calculations, the reference sequences for the *D. pseudoobscura* and *D. persimilis* genomes were both included [62],[63]. Details of diversity and divergence calculations are discussed in Text S1 (see section titled “Fine-Scale Recombination Maps: Computational Methods for Diversity and Divergence Measures”). Briefly, average pairwise diversity and divergence was calculated for 4-fold degenerate sites, focusing exclusively on unpreferred codons [64], though we obtained very similar results when using all 4-fold degenerate sites. Overall, recombination is significantly and positively associated with average pairwise diversity but not average pairwise divergence at 4-fold degenerate sites of unpreferred codons. We examined this relationship in several ways.

### Diversity, Not Divergence, Is Positively Associated with Recombination in All Intervals

We analyzed each chromosome for each uncondensed recombination map independently using a generalized linear model for diversity and a separate model for divergence (Tables S9, S10, and S11). After accounting for multiple covariates, diversity at 4-fold degenerate sites of unpreferred codons shows a significant, positive relationship with recombination, while divergence at 4-fold degenerate sites of unpreferred codons does not (Tables S9 and S10). This result is consistent for each of the three recombination maps (*D. pseudoobscura–*Flagstaff, *D. pseudoobscura*–Pikes Peak, and *D. miranda*) for both chromosome 2 and the XR chromosome arm (Tables S9 and S10). The XL chromosome arm contained too few intervals for analysis for *D. pseudoobscura–*Flagstaff. For *D. pseudoobscura*–Pikes Peak and *D. miranda*, diversity showed a significant, or nearly significant, positive relationship with recombination, while divergence did not (Table S11).

### Diversity, Not Divergence, Is Positively Associated with Recombination in Conserved Intervals

The analysis above suggests that the recombination–diversity relationship is probably the result of the effect of recombination on selection at linked sites (*sensu* [13],[18]); however, inadvertently including regions with discordant recombination rates between species in the analysis above could result in a pattern that supports this hypothesis—even when recombination is predominantly mutagenic (Figure S1). To resolve this potential bias, we restricted analysis to only regions that exhibited conserved recombination rates between all three chromosome 2 maps (*N* = 27 intervals; described above) and examined recombination in association with average pairwise *D. pseudoobscura* diversity at 4-fold degenerate sites of unpreferred codons (Table 2; Figures S7 and S8) and average pairwise *D. pseudoobscura–D. miranda* divergence at 4-fold degenerate sites of unpreferred codons (Table 3; Figures S7 and S8). The effect of recombination on diversity was significant when the analysis was restricted to only those regions with the most conserved recombination rates (quasibinomial GLM, *F* = 6.123, *p* value = 0.024), and the effect of recombination on divergence remained nonsignificant (quasibinomial GLM, *F* = 0.138, *p* value = 0.714). These regions contained only one interval within 4 Mb of the telomeric end and no intervals within 4 Mb of the centromeric end of the chromosome; thus, these results are not a function of broad-scale regional recombination rate differences across the chromosome. These results support the hypothesis that recombination affects diversity through the effect of selection on linked sites. We did not perform an analysis on conserved windows for the X chromosome, as only seven intervals were conserved within and between species.

**Table 2:** Factors affecting diversity within species at 4-fold degenerate sites for unpreferred codons using intervals with conserved recombination rate.

Factor Tested	*df*	Deviance	Residual *df*	Residual Dev.	*F*	*p* Value
Null			26	57.009		
Gene density	1	2.3190	25	54.690	2.2948	0.147171
Mutation	1	12.7343	24	41.955	12.6013	0.002289*
Recombination	1	6.1877	23	35.768	6.1231	0.023521*
GC	1	11.1854	22	24.582	11.0685	0.003751*
Gene Density×Mutation	1	2.0720	21	22.510	2.0504	0.169304
Gene Density×Recombination	1	2.8041	20	19.706	2.7748	0.113065
Mutation×GC	1	0.5488	19	19.157	0.5430	0.470669
Recombination×GC	1	0.0007	18	19.156	0.0007	0.978599

A generalized linear model with quasibinomial distribution for the fine-scale intervals on chromosome 2 with conserved recombination rates between *D. pseudoobscura* Flagstaff, *D. pseudoobscura* Pikes Peak, and *D. miranda* after correction for the global modifier. This model illustrates the relationship of average pairwise *D. pseudoobscura* diversity for 4-fold degenerate sites of unpreferred codons to various factors. Windows that were nonsignificant when analyzed with a rare events logistic regression and had an Odds Ratio between (0.62 to 1.615) across maps were considered “conserved.” For this analysis, the “neutral mutation rate” was set as the average pairwise *D. lowei*–*D. persimilis* divergence at 4-fold degenerate sites for unpreferred codons. For consistency between models, if an interaction term was significant in any of the models (see Tables 3, S9, S10, and S11), it was kept in all. Results from uncondensed chromosome 2, XL, and XR exhibit similar relationships (Tables S9, S10, and S11). An asterisk indicates significance at an α of 0.05.

**Table 3:** Factors affecting divergence between species at 4-fold degenerate sites for unpreferred codons using intervals with conserved recombination rate.

Factor Tested	*df*	Deviance	Residual *df*	Residual Dev.	*F*	*p* Value
Null			26	53.578		
Gene density	1	2.1647	25	51.414	1.1784	0.29201
Mutation	1	4.8404	24	46.573	2.6349	0.12192
Recombination	1	0.2540	23	46.319	0.1382	0.71437
GC	1	7.3218	22	38.997	3.9857	0.06124
Gene Density×Mutation	1	0.5094	21	38.488	0.2773	0.60492
Gene Density×Recombination	1	1.9069	20	36.581	1.0380	0.32178
Mutation×GC	1	0.0309	19	36.550	0.0168	0.89827
Recombination×GC	1	0.2399	18	36.310	0.1306	0.72202

The relationship of the average pairwise *D. pseudoobscura–D. miranda* divergence for 4-fold synonymous sites of unpreferred codons to various factors. All parameters are the same as Table 2.

### Recombination and Selection

To determine the impact of recombination rate on selection at linked sites in the genome, we used two generalized linear models to analyze the relationship of recombination rate and several measures that may be indicative of the efficiency of selection: (1) abundance of nonsynonymous substitutions and (2) average pairwise nucleotide diversity at 4-fold degenerate sites around nonsynonymous substitutions. We analyzed the association of recombination rate with these two measures in a generalized linear model framework to account for covariates such as gene density, GC content, and a proxy for neutral mutation rate. Biased gene conversion may influence substitution rates; thus, we controlled for GC content in all of the analyses below [7],[16],[65],[66]. We did not consider gene expression as a covariate, though some studies point to a negative relationship with recombination rate [67].

### No Correlation of Recombination With Nonsynonymous Substitution Abundance

The relationship of recombination rate to nonsynonymous substitution abundance was examined with the *D. pseudoobscura* Flagstaff fine-scale recombination maps. Nonsynonymous substitution abundance was measured as the nonsynonymous substitutions on the branch leading to *D. pseudoobscura*+*D. persimilis* as identified with PAML. The response variable was the number of nonsynonymous substitutions in each gene, and the covariates of the linear model included (1) the number of synonymous substitutions in the gene in question allowing for inclusion of genes where *K_s_* = 0 (*sensu* [50]), (2), GC content of the gene, (3) gene density of 50 kb on either side of the midpoint of the gene, and (4) average pairwise divergence at 4-fold degenerate sites of unpreferred codons between *D. persimilis* and *D. lowei* as a proxy for neutral mutation rate within the gene. We found no relationship (Table 4) between recombination and nonsynonymous substitution abundance with the fine-scale data (generalized linear model with Poisson distribution, *z* = −0.614, *p* = 0.539).

**Table 4:** Test for relationship between recombination rate and number of nonsynonymous substitutions; response: nonsynonymous substitutions along the *D. pseudoobscura+D. persimilis* lineage.

Model	Factor Tested	Estimate	SE	*z* Value	*p* Value
Fine-scale	(Intercept)	2.574891	0.207963	12.38	<0.0001*
	Synonymous	0.053427	0.001557	34.31	<0.0001*
	GC content	−4.892668	0.339146	−14.43	<0.0001*
	Gene density	0.158809	0.196072	0.81	0.418
	Neutral mutation rate	0.470959	3.417997	0.14	0.890
	Recombination	−0.015829	0.019014	0.83	0.405

A generalized linear mixed model with Poisson distribution used to compare nonsynonymous substitutions along the *D. pseudoobscura*+*D. persimilis* lineage per gene to recombination rates measured in the Flagstaff cross. Interval was included as a random effect to account for multiple genes per interval. For this analysis, the “neutral mutation rate” was set as the average pairwise *D. lowei*–*D. persimilis* divergence at 4-fold degenerate sites of unpreferred codons. An asterisk indicates significance at an α of 0.05.

### Footprints from Putative Hitchhiking May Be Slightly Larger in Low Recombination Regions

In response to selective sweeps, a trough in diversity should be visible around selected variants [30],[68]–[72]. We analyzed diversity surrounding the nonsynonymous substitutions along the lineage leading to *D. pseudoobscura*+*D. persimilis* identified by PAML. We compared the average pairwise diversity patterns at 4-fold degenerate sites surrounding these substitutions in relation to the Flagstaff recombination rate and distance in basepairs from the substitution (Text S1). In regions with high recombination rates, the footprints of selection are thought to be narrower than in regions with low recombination rates, where strong linkage between sites will create a stronger signature of sweeps [39],[69],[71],[73]. As a control, similar analyses were performed using synonymous substitutions along the *D. pseudoobscura*+*D. persimilis* lineage following [68]. Synonymous substitutions, in many cases, evolve in a more neutral fashion than nonsynonymous substitutions ([68], but see [74],[75]). In a recent genome-scale analysis conducted with data similar to what are presented here, little reduction in diversity was seen around synonymous substitutions [68]; this study instead saw an increase in diversity, which disappeared after correction for local mutation rates.

We considered 60 kb on either side of the substitution along the *D. pseudoobscura* lineage divided into 1,000 bp nonoverlapping windows (*sensu* [68]). For each 1,000 bp window, the response variable was the number of polymorphic 4-fold degenerate sites. The generalized linear model included the following covariates: (1) total 4-fold degenerate sites, (2) GC content, (3) proportion of coding bases, (4) divergence of *D. lowei*–*D. persimilis* at 4-fold degenerate sites as a proxy for neutral mutation rate, and (5) proportion of bases that were nonsynonymous substitutions. The identities of each nonsynonymous substitution were included as random effects. This generalized linear mixed model with Poisson distribution included the following factors: absolute physical distance from the substitution, fine-scale-derived estimates of recombination rate, and the interaction between these two factors. A negative interaction term means that short distances from a substitution and high recombination rates have similar effects on diversity as large distances and low recombination rates. We expect the interaction term for distance and recombination rate to be much reduced in magnitude for synonymous substitutions in comparison to the nonsynonymous analysis.

**Figure 3:**
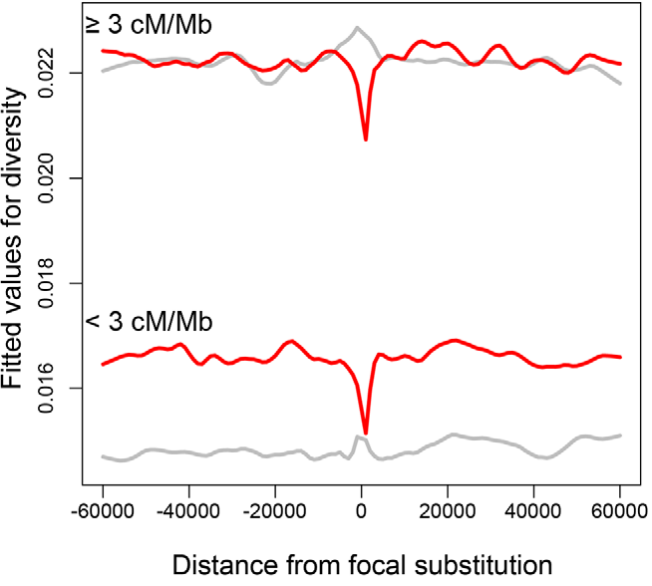
Footprints of diversity around substitutions. Fitted values for a model with nearly identical covariates as Table 5 and Table 6. Recombination rate and distance from substitution were not included in the model because they were physically plotted. Diversity of 4-fold degenerate sites was fitted as a response in the general linear model, instead of numerator (and denominator was not included in the covariates) for ease of interpretation. Center of *x*-axis represents substitutions identified along the *D. pseudoobscura*+*D. persimilis* lineage. For all graphs, a Lowess smoothing factor of 0.06 was used. Red, nonsynonymous substitutions; grey, synonymous substitutions.

We found a small but significant negative interaction term of physical distance from the nonsynonymous site and recombination rate on nucleotide diversity around nonsynonymous substitutions (Poisson GLMM, *z* = −7.52, *p*<0.001; Table 5, Figures 3 and S9). In other words, higher rates of recombination allow for recovery of diversity at shorter physical distances from the nonsynonymous site than lower recombination rates (Figure S9). In contrast, a weaker interaction was detected for the interaction of distance and recombination rate on diversity around synonymous substitutions along the *D. pseudoobscura* lineage (Poisson GLMM, *z* = −2.43, *p* = 0.015; Table 6, Figures 3 and S9). GLM plots for the very low recombination rates of <0.5 cM/Mb show wider dips in diversity (and more associated noise; Figure S9) than plots for recombination rates of >0.5 cM/Mb (Figure S9).

**Table 5:** Analysis of the diversity around nonsynonymous substitutions; response: number of 4-fold degenerate polymorphisms around nonsynonymous substitutions.

Factor Tested	Estimate	SE	*z* Value	*p* Value
Intercept	0.709549	0.006629	107.04	<0.001*
Eligible bases	0.213679	0.003627	58.91	<0.001*
GC content	0.034475	0.002937	11.74	<0.001*
Neutral mutation rate	0.102165	0.001995	51.21	<0.001*
Proportion coding	0.048260	0.003494	13.81	<0.001*
Proportion nonsynonymous	−0.089953	0.002430	−37.01	<0.001*
Absolute distance	0.032829	0.002025	16.21	<0.001*
Recombination rate	0.143129	0.006255	22.88	<0.001*
Distance×Recombination Rate	−0.014002	0.001862	−7.52	<0.001*

A generalized linear mixed model with Poisson distribution used to compare the diversity around nonsynonymous substitutions along the *D. pseudoobscura+D. persimilis* lineage in relation to recombination rates measured in the Flagstaff cross. Measures were taken 60 kb from the site in either direction (120 kb total) in nonoverlapping bins of 1,000 bp. Identity of the substitution was included as a random effect. Proportion nonsynonymous is the proportion nonsynonymous substitutions fixed along the *D. pseudoobscura+D. persimilis* lineage for each 1,000 bp window. Absolute distance is the absolute distance from the focal nonsynonymous substitution. Neutral mutation rate is the average divergence at 4-fold degenerate sites between *D. lowei*–*D. persimilis* for the 1,000 bp window. An asterisk indicates significance at an α of 0.05. All effects in the model were standardized to mean zero and unit standard deviation. Estimates given in the table must be interpreted to take this into account. Using the example of eligible bases, our model predicts that for each standard deviation increase in eligible bases above the mean, there is a 0.213679 increase in the log of the number of polymorphisms. Table S12 gives the mean and standard deviation for each factor in the model.

**Table 6:** Analysis of the diversity around synonymous substitutions; response: number of fourfold degenerate polymorphisms around synonymous substitutions.

Factor Tested	Estimate	SE	*z* Value	*p* Value
Intercept	0.603558	0.005225	115.53	<0.001*
Eligible bases	0.204713	0.002597	78.82	<0.001*
GC content	0.044745	0.002085	21.46	<0.001*
Neutral mutation rate	0.099744	0.001446	68.97	<0.001*
Proportion coding	0.047405	0.002492	19.02	<0.001*
Proportion nonsynonymous	−0.079192	0.001700	−46.58	<0.001*
Absolute distance	0.014079	0.001451	9.70	<0.001*
Recombination rate	0.200967	0.004950	40.60	<0.001*
Distance×Recombination Rate	−0.003178	0.001310	−2.43	0.0153*

A generalized linear mixed model with Poisson distribution used to compare the diversity around synonymous substitutions along the *D. pseudoobscura+D. persimilis* lineage in relation to recombination rates measured in the Flagstaff cross. All parameters and transformations were identical to those in Table 5. Table S12 gives the mean and standard deviation for each factor in the model.

Distance from a substitution had a positive, significant effect on diversity as expected if linked selection of substitutions generates a dip in diversity (Tables 5, 6, and S12). Recombination rate also had a positive, significant effect on diversity as expected, if either recombination was mutagenic or if positive/negative selection was operating on the chromosome (Tables 5, 6, and S12). The proportion of nonsynonymous substitutions around a substitution had a negative significant effect on diversity surrounding a nonsynonymous site as expected if many of these substitutions combine forces to generate stronger selective sweeps (Tables 5, 6, and S12). The interaction term pointing to deeper dips in diversity for lower recombination rates is no longer significant when examining only 5 kb or 15 kb on either side of the focal substitution (it is negative for nonsynonymous substitutions and positive for synonymous substitutions), but it is conceivable that this lack of significance represents an issue with window size or sampling.

## Discussion

Overall, our study identified both global and local differences in recombination rate between two closely related species of *Drosophila*. Aside from regions with exceptionally low recombination rates [12],[76], variation in local recombination rates between species must be accounted for prior to concluding that the association between recombination rate and diversity is probably caused by recombination modulating the effects of selection at linked sites [77]. By restricting our analysis in the *Drosophila pseudoobscura* system to only those regions with conserved recombination rates within and between species, we rejected the hypothesis that recombination rate (at the scale tested) significantly affects divergence at 4-fold degenerate sites for unpreferred codons. These results support the conclusion that recombination has a substantial impact on how selection affects diversity in the genome. Furthermore, additional analyses suggest that recombination rate variation affects the impact of Hill–Robertson effects like selective sweeps and background selection in this system.

### Ultrafine and Fine-Scale Variation in Crossover Rate in *Drosophila*

Here and in other recent work [54], we demonstrate that ultrafine-scale patterns of crossover rate (intervals spanning 20 kb) are also significantly heterogeneous in *D. pseudoobscura*. In each ultrafine region on chromosome 2, recombination rates varied by up to 6-fold (17 Mb region) over only approximately 120 kb (6 Mb region variation is 3.6-fold, and 21 Mb region variation is 5.1-fold), and ultrafine-scale maps reveal variation not detected in the fine-scale maps. This was especially apparent for the 17 Mb region, where ultrafine-scale recombination rates ranged from 3.5 to 21.2 cM/Mb, and fine-scale recombination rates in the same area ranged only from 4.4 to 5.6 cM/Mb. This heterogeneity suggests that our fine-scale measures (intervals spanning <200 kb) are averages of actual variation in recombination rate.

In humans, broad-scale variation averages over the density and intensity of ∼2 kb hotspots that occur in clusters every 60–90 kb [78],[79]. The majority of recombination occurs at these hotspots, and the majority of recombination is governed by the DNA binding protein PRDM9 and its recognition motifs in humans [17],[80]–[84]. Interestingly, several studies in different regions of the *D. melanogaster* genome indicate that linkage disequilibrium decays rapidly [37],[85]–[87], suggesting that the heterogeneity we observed in ultrafine-scale maps may not be governed by clustered hotspots similar to those in humans, or at least that a nontrivial amount of recombination may occur outside such “hotspots.”

To assess whether “hotspots” of some sort exist in *D. pseudoobscura*, genome-wide patterns of linkage decay need to be investigated or incredibly fine-scale maps (interval size <5 kb) need to be made. Such a line of inquiry would help address basic questions about the requirements for functional recombination across various taxa. For example, there are several notable differences regarding the formation and function of the synaptonemal complex and the role of double-strand breaks across taxa [88]–[93]. Furthermore, the *Drosophila* lineage completely lacks several proteins essential for generating crossovers and double-strand break repair in other organisms [89],[94]. It is likely that understanding particular sequence features associated with recombination on a kilobase scale in *Drosophila* will uncover more details about the mechanistic underpinnings of meiosis that differentiate these species and the distribution of crossovers across the genome.

### *Drosophila miranda* Has Elevated Global Recombination Rate Relative to *D. pseudoobscura*

Recombination rates at broad scales are conserved between populations and species [33],[95]–[100] (see also review in [20]). Our fine-scale data are generally consistent with these findings except that *D. pseudoobscura* has about three-fourths the rate of recombination, on average, as *D. miranda* for chromosome 2 and about three-fifths the rate of recombination of *D. miranda* on the XR chromosome arm. Notably, *D. melanogaster* has one of the lowest recombination rates in the genus, as evidence indicates that *D. mauritiana*, *D. simulans*, *D. virilis*, *D. pseudoobscura*, *D. miranda*, and *D. persimilis* all exhibit higher rates of recombination [33],[53],[99]; this should be considered when interpreting hitchhiking and linkage data from *D. melanogaster* to patterns of recombination in *Drosophila* in general.

### Recombination Prevents Diversity Erosion During Selection

Our results indicate that recombination affects diversity through mediating selection in the genome. While accounting for multiple covariates, we found no association between recombination and average pairwise divergence at 4-fold degenerate sites of unpreferred codons, and a significant, positive association of recombination with average pairwise diversity at 4-fold degenerate sites of unpreferred codons. Using data from our fine-scale maps, we ensured that recombination rates are nearly identical between the species used to generate divergence estimates; thus, we absolved a key assumption made in previous studies (see Figure S1). Data from *Drosophila* suggest both positive and negative selection are markedly less efficient in nearly nonrecombining regions of the genome [12],[47],[76],[101],[102], and a relationship of diversity but not divergence to recombination is apparent for other species of *Drosophila* [13],[33],[40],[49], mouse [36], beet [35], tomato [103],[104], *Caenorhabditis* [38], and yeast [105]. This last example is especially interesting because recombination is known to be mutagenic in yeast [24],[27], but there is a negative or absent divergence–recombination correlation [34],[105]; thus, it may be that recombination is somewhat mutagenic in many organisms, but the power of recombination to modulate the diversity eroding effects of selection likely has a much greater impact on the genome.

In other systems, the divergence–recombination association is positive, which may be interpreted as evidence that recombination is predominately mutagenic. A positive divergence–recombination association is apparent for humans [106],[107], maize [108], and in an inverted region between *D. pseudoobscura* and *D. persimilis* [25]. This association may be attributable to mutation [21], but unmeasured variables or segregating ancestral polymorphism could predispose a system to exhibiting a positive divergence–recombination relationship [34],[38]–[41]. For instance, in *C. briggsae*, segregating ancestral polymorphism leads to the signature of recombination-associated mutation (i.e., a positive divergence–recombination association), but further examination shows the majority of polymorphism heterogeneity is caused by recombination affecting the impact of selection at linked sites [38].

### Recombination Rate and Abundance of Nonsynonymous Substitutions

Since recombination probably mediates the effects of hitchhiking in our system, we sought to understand whether this hitchhiking is primarily positive or negative (background, purifying) selection and if recombination rate variation has a significant impact on the potential efficacy of selection. Evidence is emerging that in many organisms, especially those with large population sizes, selection may play a substantial role in shaping the genome [109]. For partial selfers, it seems that background selection substantially affects the genome [110]–[113], while in outcrossing species *Drosophila*, mice, and *Capsella grandiflora* a large fraction of the genome may be influenced by positive selection [40],[114]–[116]. The majority of studies find strong support that recombination can shape adaptive evolution when comparing regions of no recombination to regions with some or abundant recombination (reviewed in [7]). However, after accounting for multiple covariates in regions with detectable recombination rates, there is often very little relationship between recombination rate and the efficacy of selection [7],[12],[65].

Across chromosome 2, we found no relationship between the number of nonsynonymous substitutions and the recombination rate as measured with our fine-scale Flagstaff map. Reanalysis of the fine-scale data after removal of the first and last 3 Mb of the chromosome did not change the relationship of fine-scale recombination rate to nonsynonymous substitutions.

### Recombination Rate and Diversity Around Nonsynonymous Substitutions (GLM)

Our observation of a reduction of average pairwise diversity at 4-fold degenerate sites around nonsynonymous substitutions (Figure S9) is consistent with the idea that positive selection may have fixed many nonsynonymous substitutions along the ancestral lineage leading to *D. pseudoobscura*+*D. persimilis*, as has been argued elsewhere for other *Drosophila* species [68],[117]. While potentially less common, dips in diversity could also be caused by deleterious mutations that can get fixed by chance if deleterious selection coefficients are small enough—a situation we call “loser's luck” (Figure S10; but see [117],[118]), and theoretical investigations of entirely neutral substitutions showed that their quick fixation can also lead to dips in diversity [119]. Thus, while many of the dips in diversity we see may be caused by positive selection, both loser's luck and fixation of neutral substitutions may also contribute.

Diversity may be recovered slightly farther from a nonsynonymous substitution in areas of low recombination than in areas of high recombination, and such a relationship is not as pronounced for synonymous substitutions fixed along the lineage leading from the common ancestor of *D. pseudoobscura* and *D. persimilis* (Tables 5 and 6; Figure S9). Similarly, in *Arabidopsis*, haplotype blocks around nonsynonymous SNPs are larger than around synonymous SNPs [120]. Our data agree with theoretical expectations [69],[71] and past studies that show negative correlations of polymorphisms and nonsynonymous substitutions in *Drosophila* ([40],[68],[121],[122]; indeed, our data also show a significant negative relationship for nonsynonymous substitutions and within-species polymorphisms, generally (Tables 5 and 6). Yet the negative interaction term between recombination rate and distance from focal substitutions we observed is dependent on window size and distance from the substitution examined.

### Conclusions

Our study documented global and local differences in recombination rate between two closely related species, and these data indicate that recombination probably modulates Hill–Robertson effects in the genome, causing a positive association of diversity with recombination. While we found no overall association of recombination rate with the number of nonsynonymous substitutions at the fine scale, we found evidence for dips in diversity around nonsynonymous substitutions that are dependent on the distance from the substitution, local recombination rate, and a number of other factors. In total, our study adds to the growing literature that indicates that selection must be a ubiquitously important factor for shaping diversity across much of the genome [30],[69],[71].

## Materials and Methods

### Fine-Crossover Maps: Crosses and Technical Details

Using a backcross design, we developed two recombination maps for *D. pseudoobscura* (Flagstaff and Pikes Peak) and one recombination map for *D. miranda* (Text S1). For each cross, Duke's Genomic Analysis Facility genotyped 1,440 individual backcrossed flies for 384 line-specific SNP markers (see “SNP Development” section in Text S1) using the Illumina BeadArray platform (Illumina, San Diego, CA) [123].

### Fine-Crossover Maps: Recombination Map Construction

Recombination events were scored when an individual fly's genotype changed from heterozygous to homozygous (for the parent in the backcross) or vice versa for autosomes and when the fly's genotype changed between the possible allele combinations for the sex chromosome arms XL and XR. Double crossovers were defined as adjacent intervals with different genotypes on both sides (for instance, a single homozygote genotype call nested in a tract of heterozygote genotype calls). We deemed these as genotyping errors as crossover interference is high within 2 Mb [124] and removed the single inconsistent genotype, scoring it as missing data. CentiMorgans were defined as the number of recombination events over the total number of individuals examined for each recombination interval, and we scaled this raw measure with a correction for recombination interference [125]. Throughout the article, recombination rates are given in Kosambi centiMorgans [125] per Megabase (cM/Mb).

Approximately 1,400 backcross progeny were scored for the Pikes Peak *D. pseudoobscura* map, approximately 1,250 backcross progeny were scored for the Flagstaff *D. pseudoobscura* map, and approximately 1,170 backcross progeny were scored for the *D. miranda* map (see Table S1 for the final number of individuals, number of intervals, and size of intervals over which recombination was measured).

Physical genomic distances used to calculate centiMorgans per Megabase (cM/Mb) per interval were based on the *D. pseudoobscura* reference genome v2.6 (Flagstaff) and v2.9 (Pikes Peak, *D. miranda*). Marker order was confirmed by the R (The R Foundation for Statistical Computing 2010) package OneMap [126] using the algorithms Recombination Counting and Ordering [127] and Unidirectional Growth [128]. Onemap does not accommodate backcrossed designs for sex chromosomes; therefore, we specified an F2 intercross design in these cases. We found one small inversion in *D. miranda* relative to *D. pseudoobscura* on chromosome 2. We estimated the left breakpoint was between the markers at 10,491,527 and 10,660,216 bp, and the right breakpoint was between the markers at 13,318,705 bp and 14,068,383 bp from the telomeric end of chromosome 2. This inversion corresponds to one previously documented between *D. miranda* and *D. pseudoobscura* between markers *rosy* and *nop56* [129]. Figure S6 illustrates that recombination rate differences are probably not due to differences in gene order; thus, we used the *D. pseudoobscura* orientation for this inversion when comparing recombination between maps and excluded intervals that included the breakpoints. Confidence intervals (95%) for cM/Mb for each recombination interval were calculated by permutation [33],[54]. Confidence intervals for those intervals where we did not find a single recombinant individual were estimated from a binomial distribution—simply, we solved the equation (1−x)^N^ = 0.05, where x is the 95% upper bound of recombination frequency, and N is the number of individuals surveyed.

### Fine-Scale Recombination Maps: Defining Intervals With Conserved and Divergent Recombination

The rationale for regressing out the effect of species (when identifying conserved intervals) was to account for the globally higher recombination rate in *D. miranda* relative to *D. pseudoobscura* and to identify regions where the recombination profile overlapped (e.g., where peaks and troughs can be overlaid). To delimit conserved regions using data that have not been corrected for elevated recombination rate of *D. miranda*, one might identify a region with very similar recombination rates between *D. miranda* and *D. pseudoobscura*, but this region may be a trough in recombination rate for *D. miranda* and a peak in recombination rate for *D. pseudoobscura*. Not correcting for the global elevation of *D. miranda* may lead to falsely concluding that a region has a conserved recombination profile between two maps. Thus, we used a rare events logistic regression (Zelig package in R) between each set of condensed fine-scale recombination maps to identify regions of conserved recombination after accounting for map identity (Flagstaff–Pikes Peak, Flagstaff–*D. miranda*, Pikes Peak–*D. miranda*). The package Zelig uses the same model as a logistic regression, but it corrects for a bias that is introduced when the sample contains many more of one of the dichotomous outcomes than the other. Recombination events conditioned on the total number of observations was the response variable, and species, interval, and species-by-interval were included as factors in the model. We defined “divergent” intervals as those where tests in each interval between the species from the rare events logistic regression had a q-value of <0.05 after correction for multiple tests [59]. “Conserved intervals” were those intervals that displayed a nonsignificant difference across all three maps when analyzed with a rare events logistic regression and had an odds ratio between 0.62 and 1.615, after accounting for a species effect. We did not correct for multiple tests in defining conserved intervals. The effect size, the confidence intervals for the effect size, *p* values, and multiple-test corrected q-values are available in Datasets S1, S2, and S3.

In this way, only intervals that were conserved within and between species were delineated as conserved intervals. The final dataset used to differentiate between the mutagenic and selection hypotheses contained 27 conserved intervals on chromosome 2. We did not use the XR to differentiate between the mutagenic and selection hypotheses—of the 44 intervals condensed across three XR maps, only seven were conserved within and between species. We chose not to combine data from chromosome 2 and XR, as there is some evidence for different evolutionary patterns between autosomal and sex chromosomes in *Drosophila* [130].

### Fine-Scale Recombination Maps: Recombination, Diversity and Divergence

Details of how diversity and divergence were measured from the next generation sequencing data are given in Text S1. We analyzed the effect of recombination on diversity and divergence by applying a quasibinomial GLM as the data were overdispersed, which has several statistical properties favorable to analyzing proportions such as pairwise diversity [131],[132]. Diversity or divergence was used as a response variable by binding the number of SNP bases to the number of non-SNP, eligible bases with cbind in R. We included recombination rate, proportion of G or C bases within the recombination interval, gene density (measured as a proportion of nucleotides within the recombination interval that are coding), a proxy for neutral mutation rate (see Text S1), and interaction terms as factors in the model. See Text S1 for filtering steps that were required for a nucleotide to be considered an eligible base.

For these models, the analysis presented is restricted to those conserved, condensed intervals with highly similar recombination rates between all three maps, unless otherwise noted. This restriction removes a classic bias by requiring that the intervals have similar recombination rates between the two species compared for the divergence measures (Figure S1). Similar linear models were also analyzed using the uncondensed intervals for each of the three maps individually (Tables S9, S10, and S11). All statistics were performed in R version 2.12.1 (The R Foundation for Statistical Computing 2010) unless otherwise noted.

### Ultrafine Crossover Maps: Recombination Map Construction and Analysis

Using Flagstaff 16 and Flagstaff 14, we followed the same backcross scheme described in the section “Fine-Crossover Maps: Crosses and Technical Details.” Over 10,000 progeny from this backcross were stored in 96-well plates, frozen at −20°C and amplified for markers over these three regions. PCR products were visualized on a polyacrylamide gel using LICOR 4300 (see the section “Ultrafine Crossover Maps” in Text S1).

### Recombination and Nonsynonymous Substitutions

The number of nonsynonymous substitutions, specific to the *D. pseudoobscura*+*D. persimilis* lineage, were calculated for each gene using PAML using the resequenced genomic and reference genomic data described in Table S8 (one *D. lowei*, three *D. miranda*, three *D. persimilis*, two *D. pseudoobscura bogotana*, and 11 *D. pseudoobscura* genomes, filtered for quality as described above). We used a tree rooted with *D. lowei* and considered the branches leading to [*D. persimilis* (*D. pseudoobscura*, *D. pseudoobscura bogotana*)] to be the foreground branches (additional details in Text S1). We included *D. persimilis* a part of the foreground branch because relatively extensive interbreeding occurs between *D. pseudoobscura* and *D. persimilis* across much of the genome, aside from a few inverted regions [133]–[135].

Following [50], we used a GLMM with Poisson distribution to examine the potential for recombination rate to shape the distribution of nonsynonymous substitutions along the *D. pseudoobscura*+*D. persimilis* lineage. The model contained the following main effects: the number of silent segregating sites in each gene, GC content in each gene within Flagstaff 16, the proportion of coding bases 50 kb on either side of the gene's midpoint, weakly selected average pairwise divergence within the gene between *D. persimilis* and *D. lowei* at 4-fold degenerate sites of unpreferred codons (a proxy for neutral mutation rate), recombination rate observed for the interval containing the gene, and a random variable included to account for pseudoreplication of multiple genes per interval. The response variable was the number of nonsynonymous substitutions observed in each gene. This model construction allowed the inclusion of genes whose synonymous substitution count was zero (*sensu* [50]). The GC content from Flagstaff16 was used as this was the line used for backcrossing in the crossing scheme, and the Flagstaff map (*D. pseudoobscura*) was used in this analysis.

### Recombination and Reduction in Diversity Around Nonsynonymous Substitutions

We used 4-fold degenerate sites of unpreferred codons to measure the average levels of diversity as a function of distance from amino acid substitutions along the *D. pseudoobscura*+*D. persimilis* lineage (as identified by PAML, see above).

Generalized linear mixed models with a Poisson distribution were used to compare the diversity around nonsynonymous substitutions along the *D. pseudoobscura*+*D. persimilis* lineage in relation to distance from the site and recombination rates measured in the Flagstaff cross. Measures of diversity at 4-fold degenerate sites were taken 60 kb (*sensu* [68]) from the site in either direction (120 kb total) with nonoverlapping bins of 1,000 bp. The random effects of identities of each substitution were estimated. We included as covariates (1) divergence between *D. persimilis* and *D. lowei* at 4-fold degenerate sites (a proxy for neutral mutation rate), (2) proportion of bases that were either G or C in Flagstaff 16 within the 1,000 bp window, (3) proportion of codons that were nonsynonymous substitutions within the 1,000 bp window, and (4) proportion of bases that were coding over each 1,000 bp window. The absolute value of the distance from the site and local recombination rate (at the particular nonsynonymous substitution) were included in the model as well as the interaction between distance and recombination rate. All effects in the model were standardized to mean zero and unit standard deviation. As a control, similar analyses were performed using synonymous substitutions along the *D. pseudoobscura*+*D. persimilis* lineage. Synonymous substitutions should evolve in a more neutral fashion; thus, less of an interaction between distance and recombination rate is expected. Any 1,000 bp window with less than 75 eligible, 4-fold degenerate sites was excluded from the analysis. Any nonsynonymous or nonsynonymous changes with less than 10 windows were excluded from the analysis. For the 60 kb analysis, after all filtering steps, our data consisted of 4,338 nonsynonymous and 8,670 synonymous substitutions along the *D. pseudoobscura*+*D. persimilis* lineage on chromosome 2. Four-fold degenerate sites were used here, rather than 4-fold degenerate sites at unpreferred codons, because too little data were available in each 1,000 bp nonoverlapping window.

## Supporting Information

Dataset S1 Recombination estimates and confidence intervals.
(XLSX)

Dataset S2 Results of rare-events logistic regression for pairwise map comparisons; intervals condensed across two maps.
(XLS)

Dataset S3 Results of rare-events logistic regression for pairwise map comparisons; intervals condensed across all three maps.
(XLS)

Figure S1 Expected relationships of alternative hypotheses. Expectation of the relationship between divergence and recombination rate if the recombination–diversity positive correlation is the result of recombination being mostly mutagenic or the result of recombination's effect on selection at linked sites. (A) Neutral mutations should accumulate at the same rate within and between species; thus, if recombination is mutagenic, diversity and divergence will have the same pattern, while (B) background selection and selective sweeps are not expected to produce a consistent trend for recombination and between-species divergence. (C) Recombination rate differences between species can lead to incorrect conclusions. Illustration of the importance of measuring recombination rate in both species that are used to generate divergence measures in order to reject the hypothesis that mutagenic recombination drives the recombination rate–diversity association.
(TIF)

Figure S2 Relationships of study species. Reconstructed phylogeny for the mitochondrial gene *cytochrome oxidase II*. Branch lengths are consistent with [140].
(TIF)

Figure S3 Fine-scale recombination rates on XR. Uncondensed raw recombination rates and 95% CI for intervals along the XR. Top, *D. pseudoobscura* Flagstaff map; middle, *D. pseudoobscura* Pikes Peak map; bottom, *D. miranda*. Recombination rate is given in Kosambi centiMorgans per Megabase.
(TIF)

Figure S4 Fine-scale recombination rates on XL. Uncondensed raw recombination rates and 95% CI for intervals along the XL. Top, *D. pseudoobscura* Pikes Peak map; Bottom, *D. miranda*. Recombination rate is given in Kosambi centiMorgans per Megabase. Flagstaff is not shown, because it was surveyed at a much more coarse level (intervals 2.4 kb on average) and was relatively uninformative.
(TIF)

Figure S5 Fine-scale recombination rates for condensed intervals without and with global modifier correction. Plot of fine-scale recombination data across chromosome 2. Green line, *D. miranda*; purple, *D. pseudoobscura* Pikes Peak; blue, *D. pseudoobscura* Flagstaff. Intervals (*N* = 97) are condensed across maps to include only markers with close positions across all three maps. Top, *D. miranda* exhibits globally higher recombination rates (1.283-fold higher Odds Ratio) than either *D. pseudoobscura*. Bottom, *D. miranda* recombination rate adjusted for this global difference (i.e., original data ×0.763). Recombination rate is given in Kosambi centiMorgans per Megabase.
(TIF)

Figure S6 Fine-scale recombination rates for condensed intervals with alternate orientations for *Drosophila miranda* chromosome 2 inversion. We estimated that one breakpoint of the inversion was between the markers at 10.491 Mb and 10.660 Mb, and the other breakpoint was between the markers at 13.318 Mb and 14.068 Mb from the telomeric end (0 Mb) of chromosome 2. In Figure S5, the inverted region is shown with the sequence in relation to the *D. pseudoobscura* chromosome 2 arrangement in both top and bottom panels. Green line, *D. miranda*; purple, *D. pseudoobscura* Pikes Peak; blue, *D. pseudoobscura* Flagstaff. Top, *D. miranda* inversion in its correct orientation. Recombination rates are not corrected for the globally higher recombination rates in *D. miranda* relative to *D. pseudoobscura*. Bottom, *D. miranda* inversion is oriented relative to *D. pseudoobscura* arrangement, and recombination rate of *D. miranda* is adjusted for the global elevation relative to *D. pseudoobscura*. Recombination rate is given in Kosambi centiMorgans per Megabase. Any discordant and conserved regions are likely the result of sequence and not position on the chromosome.
(TIF)

Figure S7 No divergence–recombination correlation. Relationship of recombination rate to diversity (filled circles, solid line, *t* = 1.3398, *df* = 25, *p* value = 0.192) and divergence (open circles, dotted line, *t* = 0.4559, *df* = 25, *p* value = 0.6524) for fine-scale regions with conserved recombination between *D. pseudoobscura*–*D. miranda*. Divergence, y = 0.0001x+0.0151; Diversity, y = 0.0002x+0.0078. Figure S8 contains the same graph without the outliers at the highest recombination rate.
(TIF)

Figure S8 Identical to Figure S7 excluding high-recombination outliers. Relationship of recombination rate to diversity (filled circles, solid line, *t* = 2.2158, *df* = 24, *p* value = 0.0364) and divergence (open circles, dotted line, *t* = 1.3257, *df* = 24, *p* value = 0.1974) for fine-scale regions with conserved recombination between *D. pseudoobscura*–*D. miranda*. Divergence between *D. miranda*–*D. pseudoobscura* has no significant relationship with recombination. This graph is identical to Figure S7, except the outliers at the highest recombination rates are removed.
(TIF)

Figure S9 Footprints in diversity around substitutions. Fitted values for a model with nearly identical covariates as Table 5 and Table 6. Diversity of 4-fold degenerate sites was fitted as a response in the general linear model, instead of numerator (and denominator was not included in the covariates) for ease of interpretation. Recombination and distance from the substitution are physically plotted and so were not included in the model. (A) Center of *x*-axis represents nonsynonymous substitutions identified along the *D. pseudoobscura*+*D. persimilis* lineage. (B) Center of *x*-axis represents synonymous substitutions identified along the *D. pseudoobscura*+*D. persimilis* lineage. For all graphs, a Lowess smoothing factor of 0.06 was used. Line colors represent the same recombination rates in (B) as what is denoted in (A).
(TIF)

Figure S10 The small band of mutational effects, where “loser's luck” can lead to the fixation of slightly deleterious mutations. This example is based on an assumed effective population size of *N_e_* = *N* = 10^6^. (A) Fixation times and overview. Black lines, the expected time to fixation is the same for advantageous and deleterious mutations (the two lines computed separately for both are printed on top of each other and are indistinguishable); blue line, ratio of fixation times (advantageous/neutral); red line, ratio of fixation probabilities (advantageous/deleterious). The expected time to fixation for neutral mutations is 4 *N_e_* generations with a standard deviation of 2.15 *N_e_*, which is on the order of the fixation time [136],[137]. Thus, neutral mutations can also lead to dips in diversity [119],[138]. (B) The fixation probability for advantageous (blue) and deleterious (black) alleles starts to quickly diverge after passing the border of neutrality (defined as *N_e_s* = 0.5 and marked with a vertical grey line). All lines were computed for a new mutation of the specified genic selection coefficient using single locus population genetics diffusion theory described elsewhere [136],[139]. Loser's luck can lead to the fixation of slightly deleterious mutations; this results in a slightly reduced expected time to fixation (see marked area).
(TIF)

Table S1 All intervals for which recombination was measured using a backcrossing scheme starting with two inbred lines. Three separate backcrosses and recombination maps were made. The first used two inbred lines of *Drosophila pseudoobscura* that were homozygous for the Arrowhead inversion on chromosome 3 (Flagstaff). The second used two inbred lines of *D. pseudoobscura* that were homozygous for the Pikes Peak inversion on chromosome 3 (Pikes Peak), and the third used two inbred lines of *D. miranda*. Median size is listed below the mean interval size for each category. Interval sizes are given in kb. CT intervals refer to intervals near the centromere or telomere. These markers were designed to span larger intervals because previous work indicated that recombination is less frequent near the centromere or telomere. N, average number of individuals scored with double crossovers removed.
(PDF)

Table S2 Uncondensed intervals over which recombination was measured across three recombination maps (*D. pseudoobscura*–Pikes Peak, *D. pseudoobscura*–Flagstaff, *D. miranda*). For “crossovers per individual,” the numbers given are mean/median/mode. “Total Mb covered” is the total distance spanned by the markers used to measure recombination.
(PDF)

Table S3 Recombination rate for regions of chromosome 2 in Kosambi cM/Mb. The telomere was defined as the end of the chromosome to 2.977 Mb. Centromere was defined as the 27.056 Mb end of chromosome. For the Pikes Peak telomere, the first marker was at 838 bp, whereas for Flagstaff and *D. miranda* maps, the first markers were at 0.483 Mb and 0.484 Mb, respectively. Using a marker at 0.483 Mb as a start point for Pikes Peak, results in an average telomeric recombination rate of 1.248 Kosambi cM/Mb.
(PDF)

Table S4 Chromosome 2 primers used for ultrafine recombination map of Flagstaff 16 backcrossed progeny. All primers amplify loci that differentiate between Flagstaff 16 and Flagstaff 14 by an indel. The location listed is relative to the reference genome of *Drosophila pseudoobscura* v2.9. Indel, putative indel size in bop; line, line in which putative indel is found.
(PDF)

Table S5 Measures of ultrafine-scale recombination rate and 95% confidence intervals (low cM/Mb and high cM/Mb) for three regions on chromosome 2 constructed from Flagstaff backcrossed progeny described in the text. Values of 0 cM/Mb for the low confidence intervals were used in place of the negative output by the simulations used to calculate the confidence interval. Primers used for ultrafine recombination map are given in Table S4. The marker location listed is relative to the reference genome of *Drosophila pseudoobscura* v2.9. Interval sizes were confirmed with 76 bp and 9 kb insert mate-paired Illumina reads. Total, total number of individual F2 backcross progeny that were genotyped.
(PDF)

Table S6 Condensed conserved interval information for chromosome 2. (A) Numbers and size of the condensed, conserved intervals between all three maps for chromosome 2. Only chromosome 2 conserved intervals were used for downstream analysis. (B) Average physical differences of marker placement between three maps for the condensed, conserved intervals used in the analysis. All values given are numbers of nucleotides based on the *D. pseudoobscura* reference genome v2.9.
(PDF)

Table S7 Conserved, condensed intervals. Intervals displayed nonsignificant difference across all three maps when analyzed with a rare events logistic regression and had an Odds Ratio between 0.62 and 1.615 after accounting for the effect of map. Interval windows for each map are given in bp in relation to the reference genome for *Drosophila pseudoobscura* v2.9. *miranda*, *D. miranda* recombination rate; PP, Pikes Peak recombination rate; Flagstaff, Flagstaff recombination rate. The recombination rates given in the table have not been corrected for a global modifier.
(PDF)

Table S8 Amount of sequence data obtained for resequenced *Drosophila* genomes. PE, paired-end. *Total number of reads and base pairs is double the amount listed if “PE” follows run type or if the run type was mate-paired. All data were submitted to the sequence read archive. Accession numbers SRA044960.1, SRA044955.2, and SRA044956.1.
(PDF)

Table S9 Quasibinomial linear model illustrating the relationship of within-species diversity and between-species divergence at 4-fold degenerate sites of unpreferred codons to various factors for chromosome 2. Neutral mutation rate was the *D. persimilis*–*D. lowei* divergence at 4-fold degenerate sites of unpreferred codons. For consistency, interaction terms significant in any of the models were kept in all. Intervals were not condensed across maps and recombination rate was not corrected for a global modifier.
(PDF)

Table S10 Quasibinomial linear model illustrating the relationship of within-species diversity and between-species divergence at 4-fold degenerate sites of unpreferred codons to various factors for the XR chromosome arm. Neutral mutation rate was the *D. persimilis*–*D. lowei* divergence at 4-fold degenerate sites for unpreferred codons sites. For consistency, interaction terms significant in any of the models were kept in all. Intervals were not condensed across maps, and recombination rate was not corrected for a global modifier.
(PDF)

Table S11 Quasibinomial linear model illustrating the relationship of within-species diversity and between-species divergence at 4-fold degenerate sites of unpreferred codons to various factors for the XL chromosome arm. Neutral mutation rate was the *D. persimilis*–*D. lowei* divergence at 4-fold degenerate sites of unpreferred codons. For consistency, interaction terms significant in any of the models were kept in all. Intervals were not condensed across maps, and recombination rate was not corrected for a global modifier. Only *D. miranda* and *D. pseudoobscura*–Pikes Peak are given because there were too few intervals for the *D. pseudoobscura*–Flagstaff map to perform the analysis.
(PDF)

Table S12 Mean and standard deviation for each factor in the models presented in Table 5 and Table 6.
(PDF)

Text S1 Supporting information and methods.
(DOCX)

## Abbreviations

GLM: Generalized Linear Model
cM/Mb: centiMorgans per Megabase
RH: recombination mediates selection at linked sites
