# Supplementary figures and images for: Recombination Modulates How Selection Affects Linked Sites in Drosophila
Source: PLoS Biol. 2012 Nov 13;10(11):e1001422. doi: 10.1371/journal.pbio.1001422 (PMC3496668; doi:10.1371/journal.pbio.1001422)

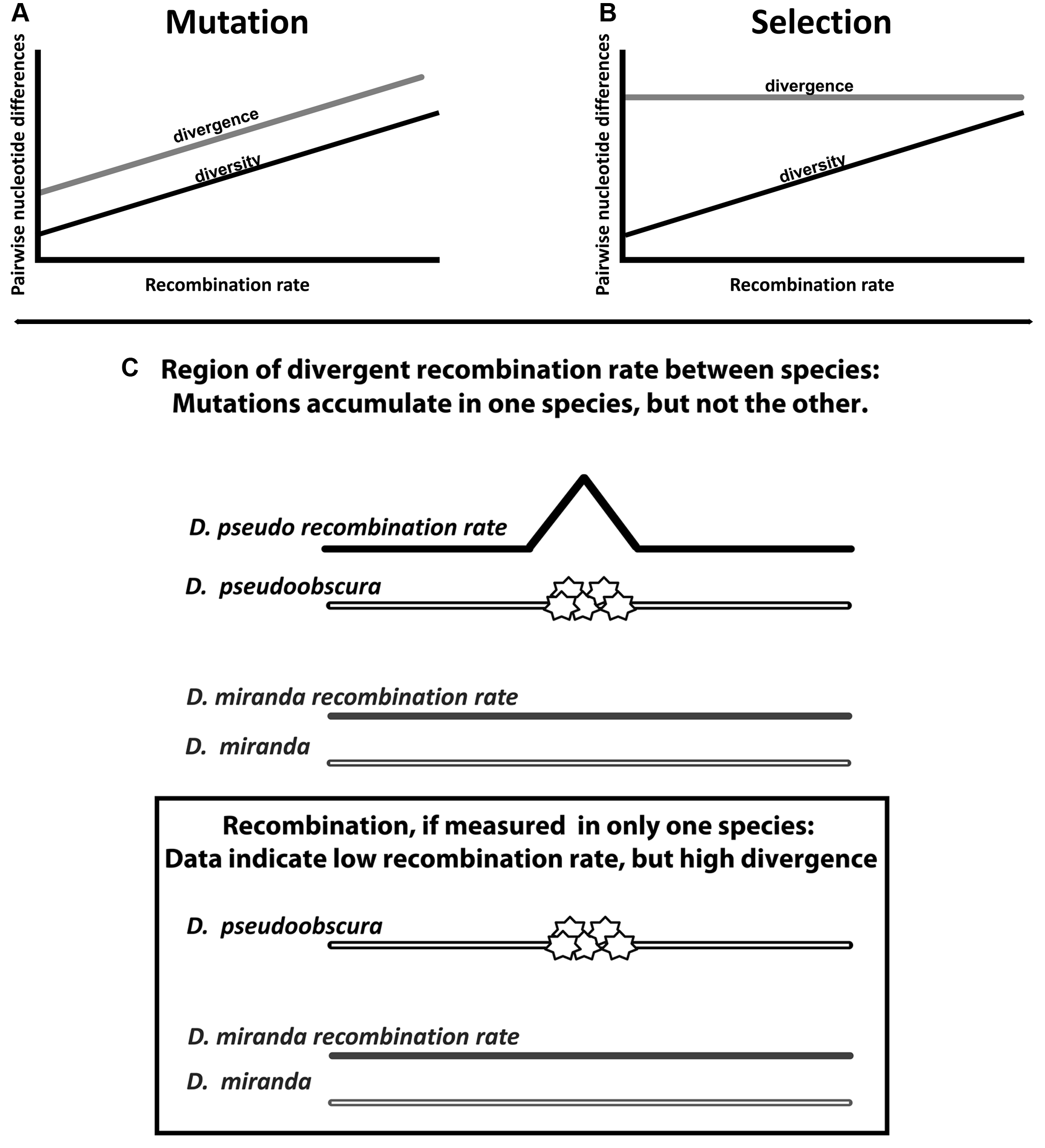

Supplement: Figure S1 — Expected relationships of alternative hypotheses. Expectation of the relationship between divergence and recombination rate if the recombination–diversity positive correlation is the result of recombination being mostly mutagenic or the result of recombination's effect on selection at linked sites. (A) Neutral mutations should accumulate at the same rate within and between species; thus, if recombination is mutagenic, diversity and divergence will have the same pattern, while (B) background selection and selective sweeps are not expected to produce a consistent trend for recombination and between-species divergence. (C) Recombination rate differences between species can lead to incorrect conclusions. Illustration of the importance of measuring recombination rate in both species that are used to generate divergence measures in order to reject the hypothesis that mutagenic recombination drives the recombination rate–diversity association. (TIF) [file pbio.1001422.s004.tif]

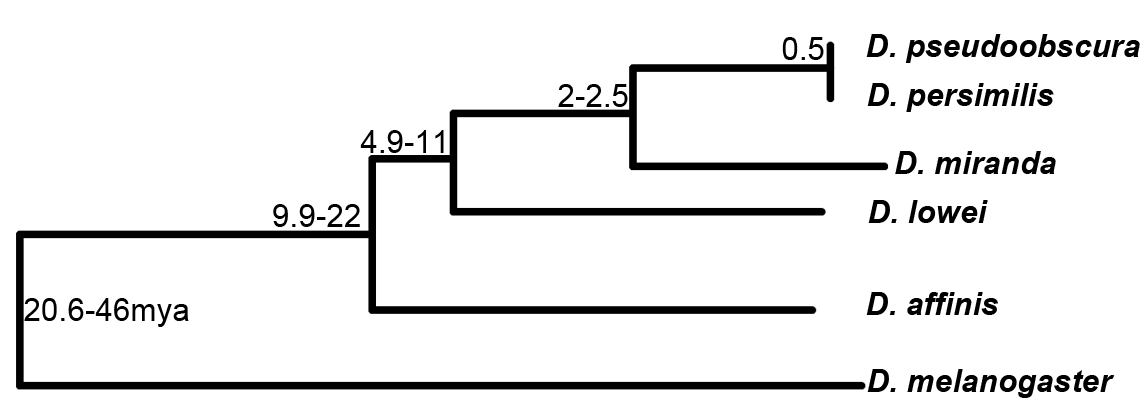

Supplement: Figure S2 — Relationships of study species. Reconstructed phylogeny for the mitochondrial gene cytochrome oxidase II. Branch lengths are consistent with [140]. (TIF) [file pbio.1001422.s005.tif]

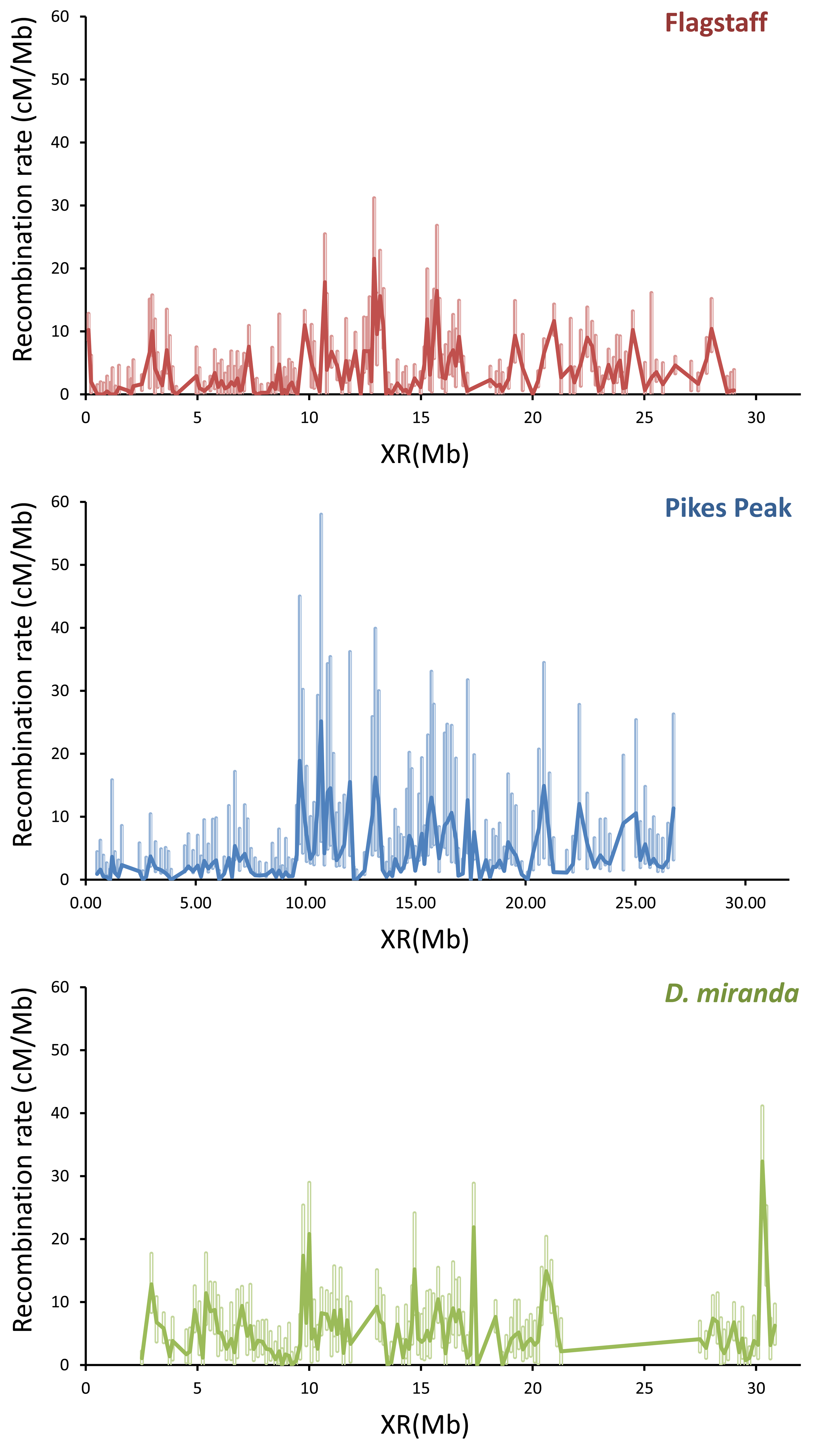

Supplement: Figure S3 — Fine-scale recombination rates on XR. Uncondensed raw recombination rates and 95% CI for intervals along the XR. Top, D. pseudoobscura Flagstaff map; middle, D. pseudoobscura Pikes Peak map; bottom, D. miranda. Recombination rate is given in Kosambi centiMorgans per Megabase. (TIF) [file pbio.1001422.s006.tif]

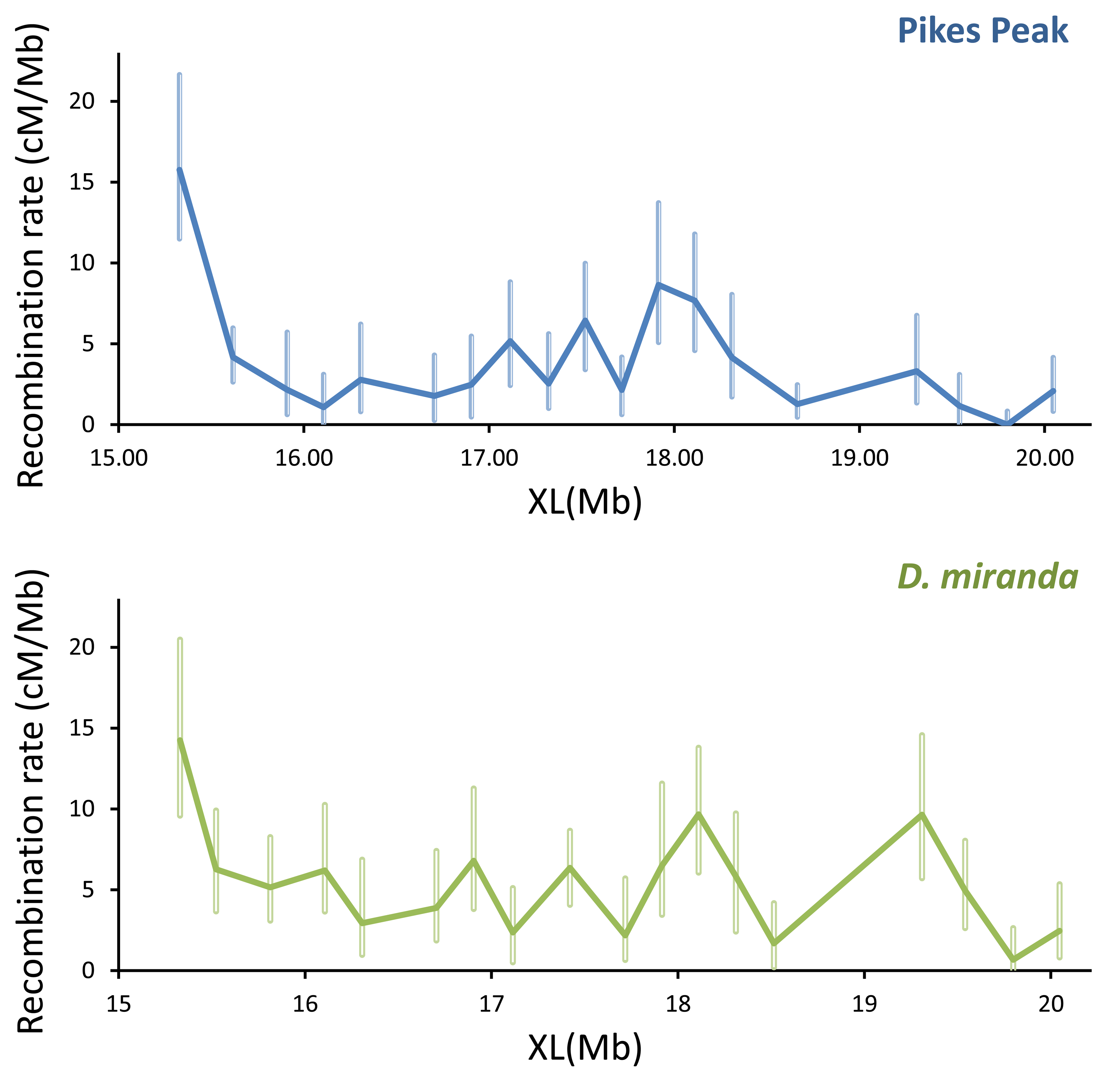

Supplement: Figure S4 — Fine-scale recombination rates on XL. Uncondensed raw recombination rates and 95% CI for intervals along the XL. Top, D. pseudoobscura Pikes Peak map; Bottom, D. miranda. Recombination rate is given in Kosambi centiMorgans per Megabase. Flagstaff is not shown, because it was surveyed at a much more coarse level (intervals 2.4 kb on average) and was relatively uninformative. (TIF) [file pbio.1001422.s007.tif]

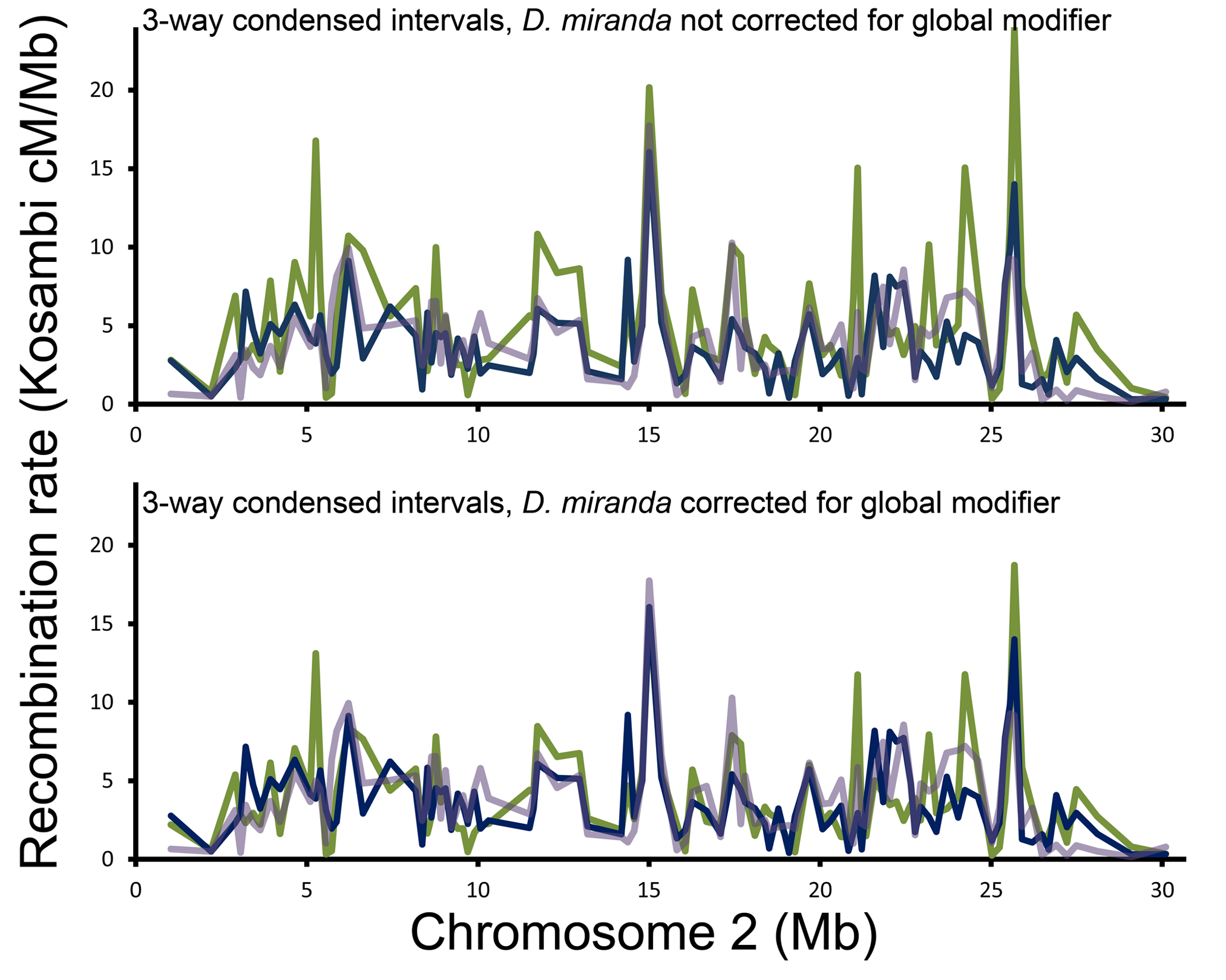

Supplement: Figure S5 — Fine-scale recombination rates for condensed intervals without and with global modifier correction. Plot of fine-scale recombination data across chromosome 2. Green line, D. miranda; purple, D. pseudoobscura Pikes Peak; blue, D. pseudoobscura Flagstaff. Intervals (N = 97) are condensed across maps to include only markers with close positions across all three maps. Top, D. miranda exhibits globally higher recombination rates (1.283-fold higher Odds Ratio) than either D. pseudoobscura. Bottom, D. miranda recombination rate adjusted for this global difference (i.e., original data ×0.763). Recombination rate is given in Kosambi centiMorgans per Megabase. (TIF) [file pbio.1001422.s008.tif]

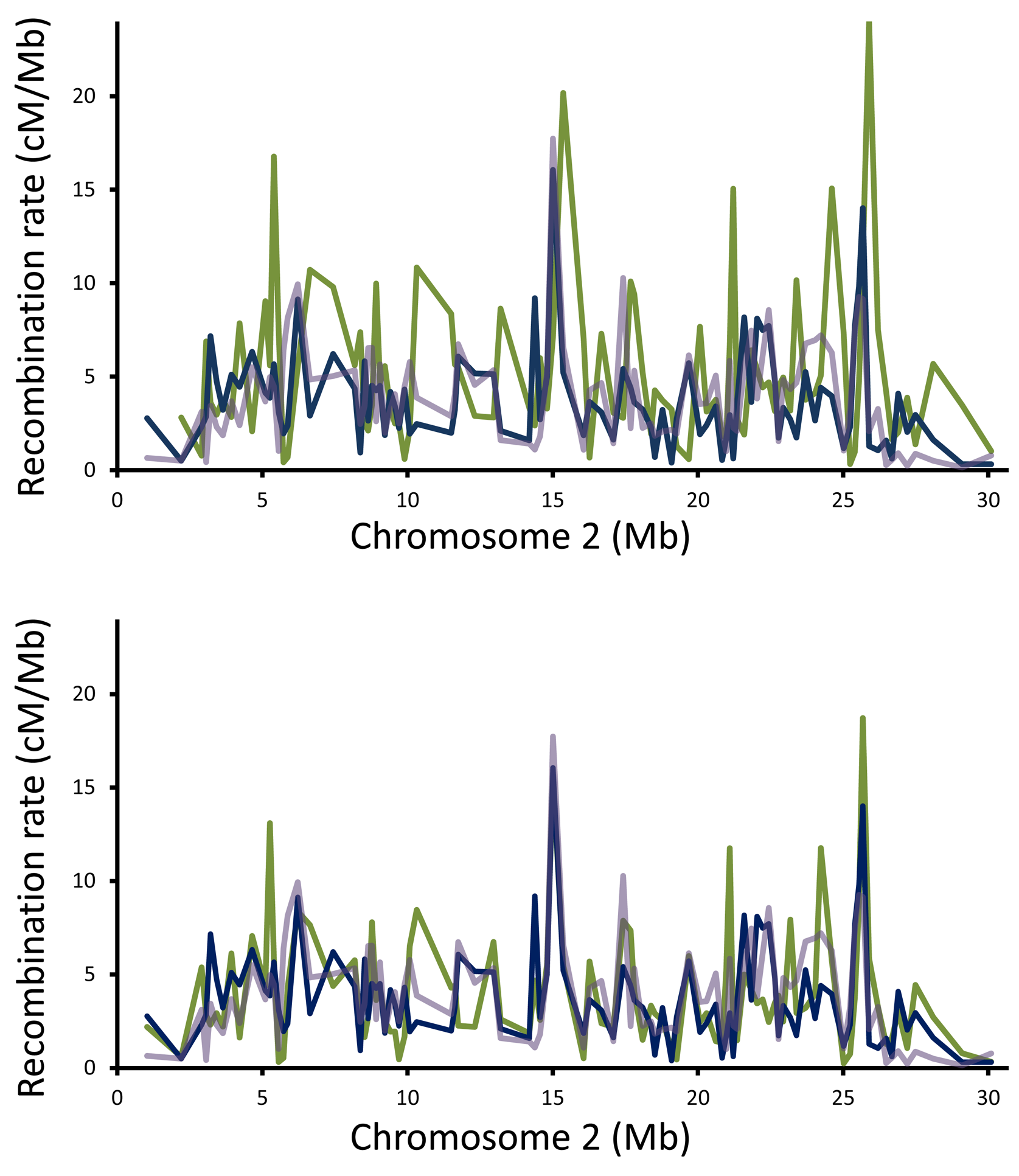

Supplement: Figure S6 — Fine-scale recombination rates for condensed intervals with alternate orientations for Drosophila miranda chromosome 2 inversion. We estimated that one breakpoint of the inversion was between the markers at 10.491 Mb and 10.660 Mb, and the other breakpoint was between the markers at 13.318 Mb and 14.068 Mb from the telomeric end (0 Mb) of chromosome 2. In Figure S5, the inverted region is shown with the sequence in relation to the D. pseudoobscura chromosome 2 arrangement in both top and bottom panels. Green line, D. miranda; purple, D. pseudoobscura Pikes Peak; blue, D. pseudoobscura Flagstaff. Top, D. miranda inversion in its correct orientation. Recombination rates are not corrected for the globally higher recombination rates in D. miranda relative to D. pseudoobscura. Bottom, D. miranda inversion is oriented relative to D. pseudoobscura arrangement, and recombination rate of D. miranda is adjusted for the global elevation relative to D. pseudoobscura. Recombination rate is given in Kosambi centiMorgans per Megabase. Any discordant and conserved regions are likely the result of sequence and not position on the chromosome. (TIF) [file pbio.1001422.s009.tif]

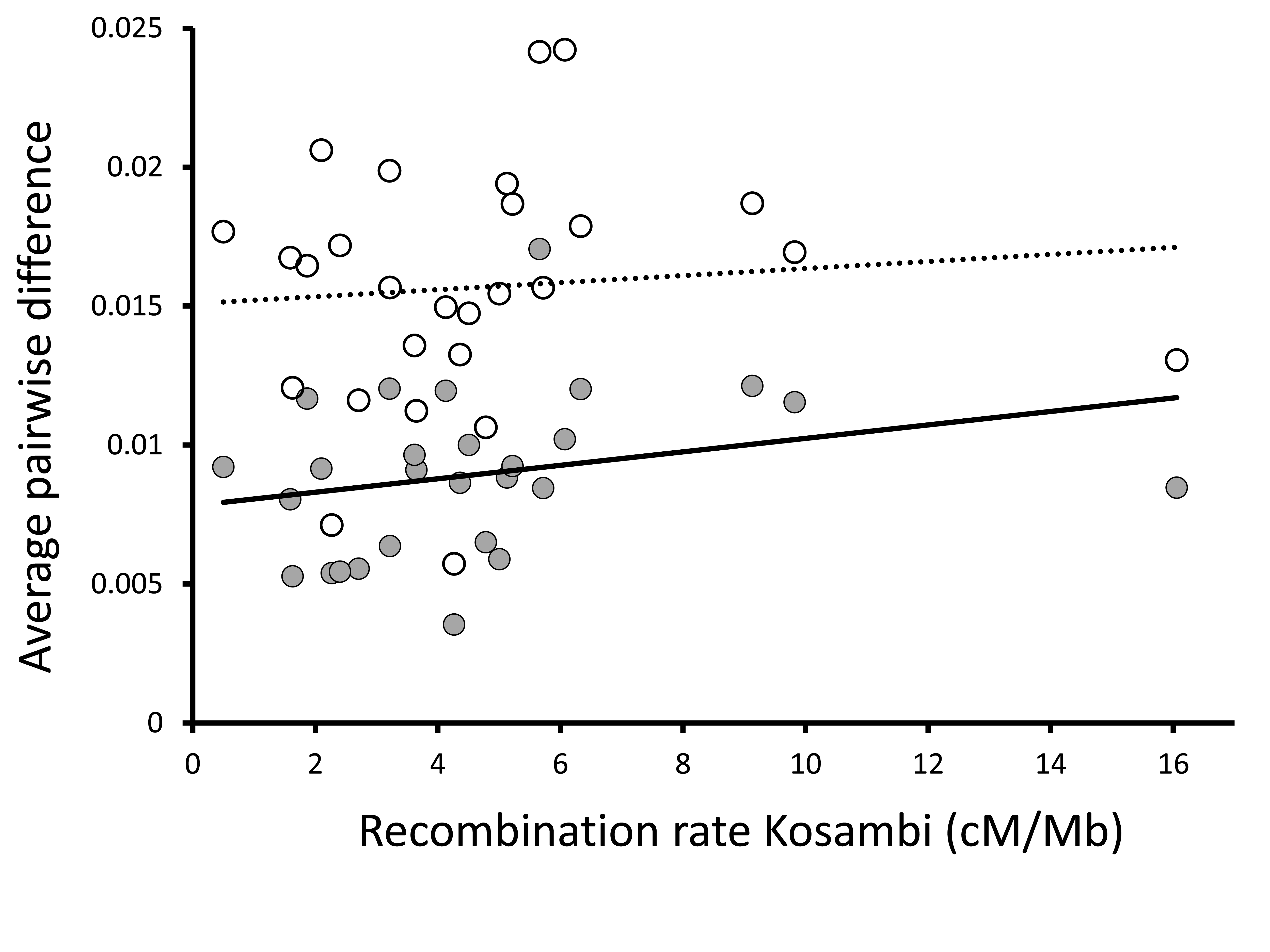

Supplement: Figure S7 — No divergence–recombination correlation. Relationship of recombination rate to diversity (filled circles, solid line, t = 1.3398, df = 25, p value = 0.192) and divergence (open circles, dotted line, t = 0.4559, df = 25, p value = 0.6524) for fine-scale regions with conserved recombination between D. pseudoobscura–D. miranda. Divergence, y = 0.0001x+0.0151; Diversity, y = 0.0002x+0.0078. Figure S8 contains the same graph without the outliers at the highest recombination rate. (TIF) [file pbio.1001422.s010.tif]

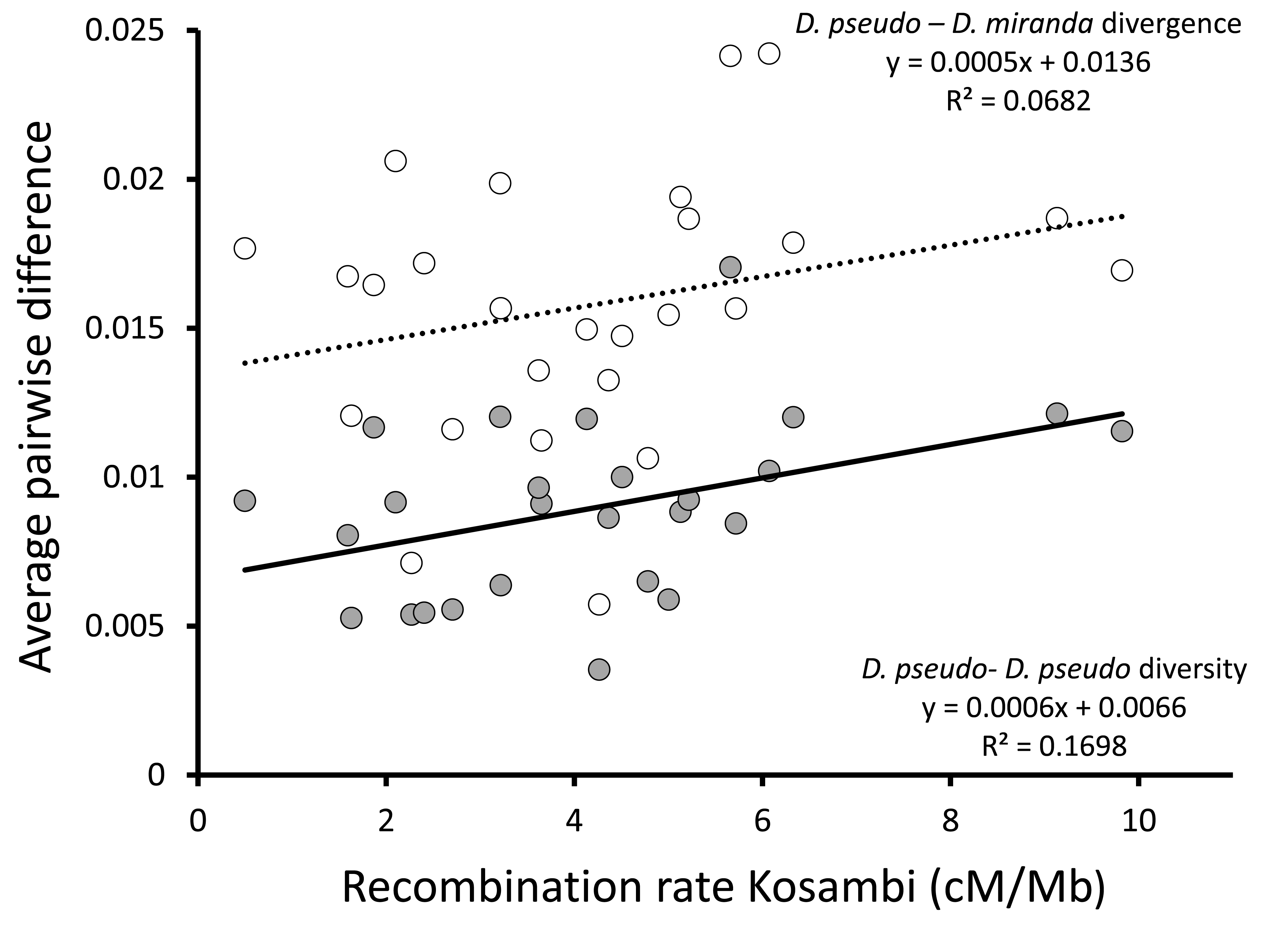

Supplement: Figure S8 — Identical to Figure S7 excluding high-recombination outliers. Relationship of recombination rate to diversity (filled circles, solid line, t = 2.2158, df = 24, p value = 0.0364) and divergence (open circles, dotted line, t = 1.3257, df = 24, p value = 0.1974) for fine-scale regions with conserved recombination between D. pseudoobscura–D. miranda. Divergence between D. miranda–D. pseudoobscura has no significant relationship with recombination. This graph is identical to Figure S7, except the outliers at the highest recombination rates are removed. (TIF) [file pbio.1001422.s011.tif]

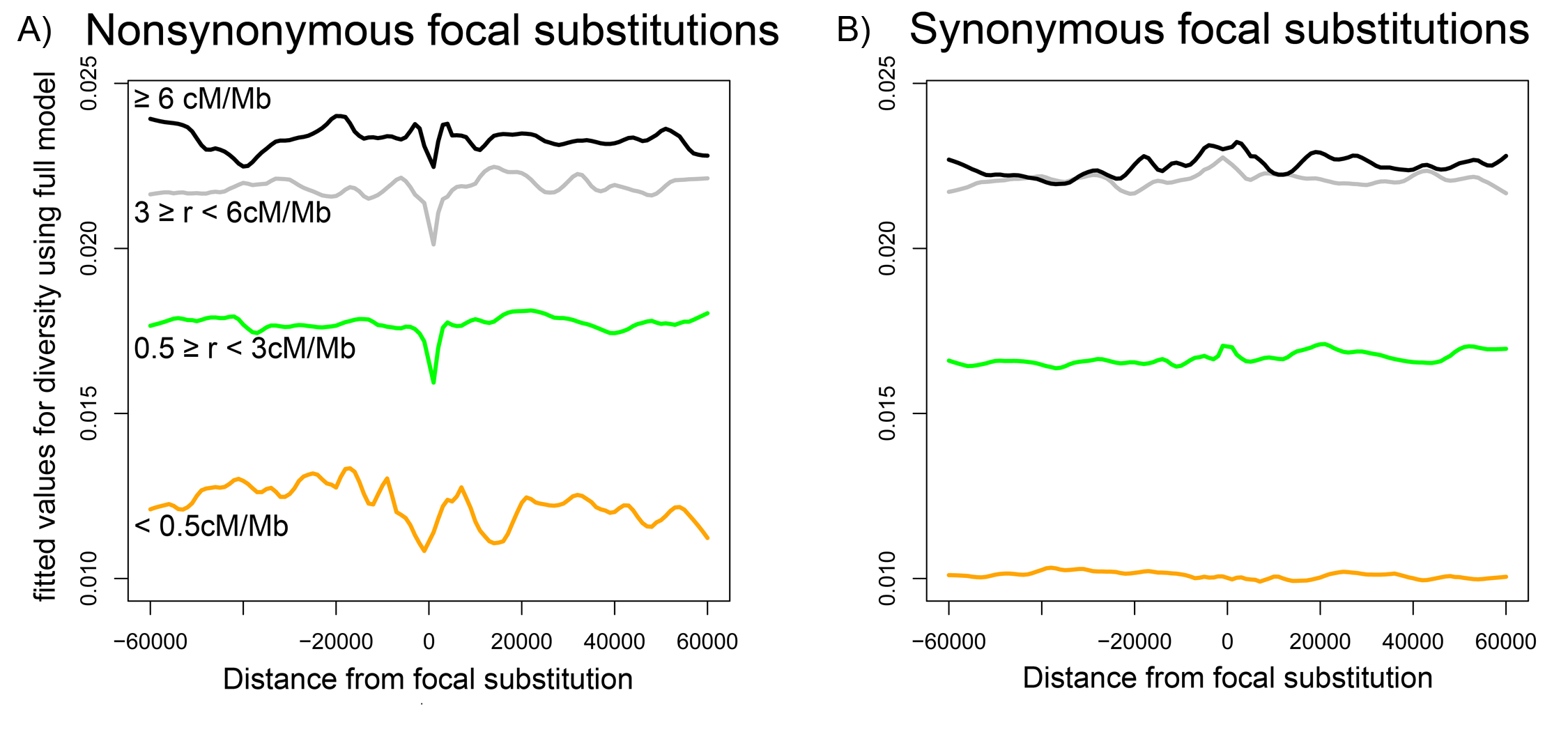

Supplement: Figure S9 — Footprints in diversity around substitutions. Fitted values for a model with nearly identical covariates as Table 5 and Table 6. Diversity of 4-fold degenerate sites was fitted as a response in the general linear model, instead of numerator (and denominator was not included in the covariates) for ease of interpretation. Recombination and distance from the substitution are physically plotted and so were not included in the model. (A) Center of x-axis represents nonsynonymous substitutions identified along the D. pseudoobscura+D. persimilis lineage. (B) Center of x-axis represents synonymous substitutions identified along the D. pseudoobscura+D. persimilis lineage. For all graphs, a Lowess smoothing factor of 0.06 was used. Line colors represent the same recombination rates in (B) as what is denoted in (A). (TIF) [file pbio.1001422.s012.tif]

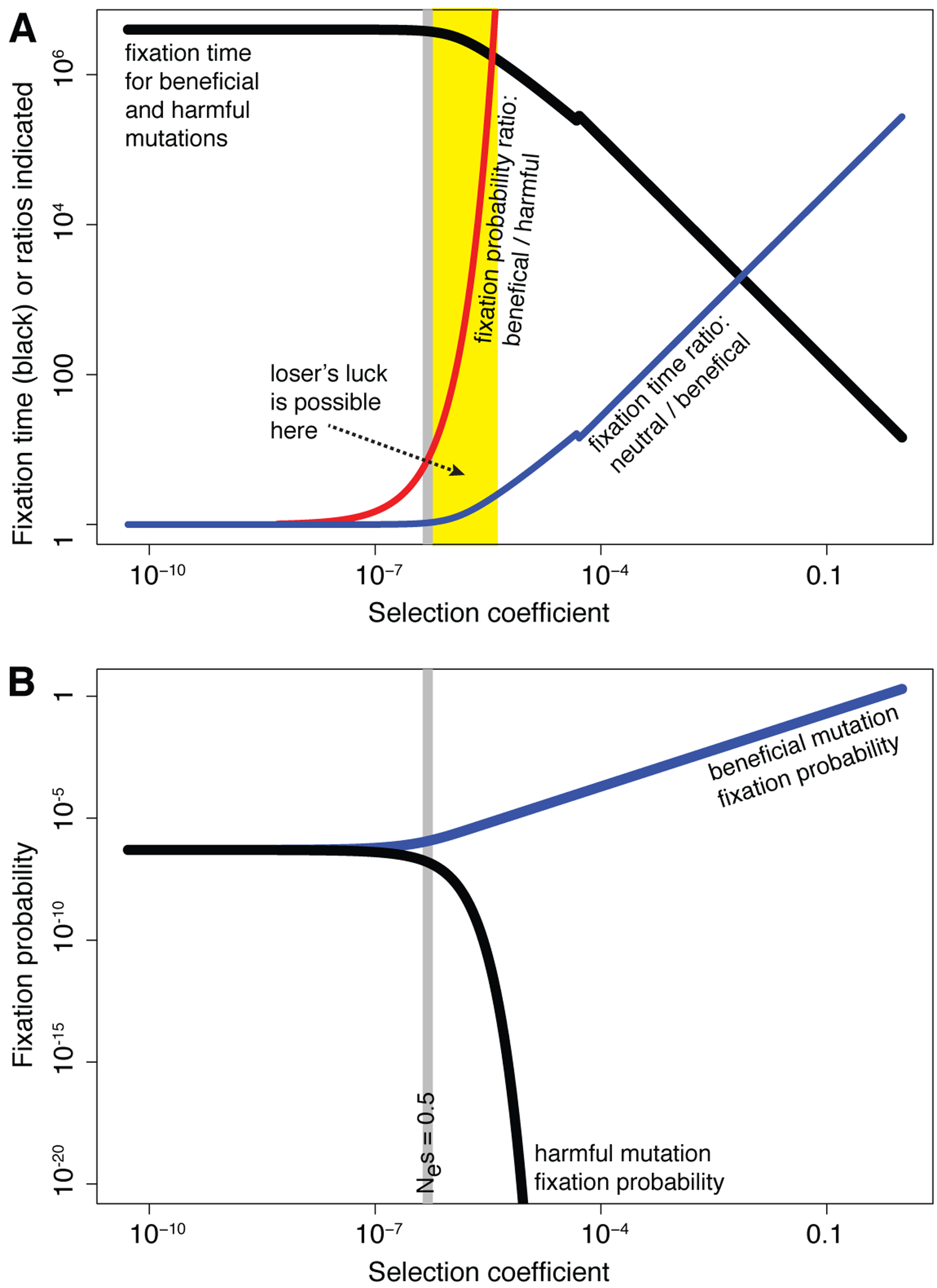

Supplement: Figure S10 — The small band of mutational effects, where “loser's luck” can lead to the fixation of slightly deleterious mutations. This example is based on an assumed effective population size of Ne = N = 106. (A) Fixation times and overview. Black lines, the expected time to fixation is the same for advantageous and deleterious mutations (the two lines computed separately for both are printed on top of each other and are indistinguishable); blue line, ratio of fixation times (advantageous/neutral); red line, ratio of fixation probabilities (advantageous/deleterious). The expected time to fixation for neutral mutations is 4 Ne generations with a standard deviation of 2.15 Ne, which is on the order of the fixation time [136],[137]. Thus, neutral mutations can also lead to dips in diversity [119],[138]. (B) The fixation probability for advantageous (blue) and deleterious (black) alleles starts to quickly diverge after passing the border of neutrality (defined as Nes = 0.5 and marked with a vertical grey line). All lines were computed for a new mutation of the specified genic selection coefficient using single locus population genetics diffusion theory described elsewhere [136],[139]. Loser's luck can lead to the fixation of slightly deleterious mutations; this results in a slightly reduced expected time to fixation (see marked area). (TIF) [file pbio.1001422.s013.tif]
