# Supplementary material for: Recombination Modulates How Selection Affects Linked Sites in Drosophila
Source: PLoS Biol. 2012 Nov 13;10(11):e1001422. doi: 10.1371/journal.pbio.1001422 (PMC3496668; doi:10.1371/journal.pbio.1001422)
Supplement: Table S1 — All intervals for which recombination was measured using a backcrossing scheme starting with two inbred lines. Three separate backcrosses and recombination maps were made. The first used two inbred lines of Drosophila pseudoobscura that were homozygous for the Arrowhead inversion on chromosome 3 (Flagstaff). The second used two inbred lines of D. pseudoobscura that were homozygous for the Pikes Peak inversion on chromosome 3 (Pikes Peak), and the third used two inbred lines of D. miranda. Median size is listed below the mean interval size for each category. Interval sizes are given in kb. CT intervals refer to intervals near the centromere or telomere. These markers were designed to span larger intervals because previous work indicated that recombination is less frequent near the centromere or telomere. N, average number of individuals scored with double crossovers removed. (PDF) [file pbio.1001422.s014.pdf]

	N	Central intervals	Mean interval size (range)	CT intervals	Mean CT interval size
D. pseudoobscura — Flagstaff					
Chr2	1256	135	183.49 kb (33.99-672.61 kb)	5	1,069.59 (984.55-1,269.71)
			median = 141.2		median = 1,012.30
XL	1253	5	2,409.56 (1,691.92-3,465.99)		
			median = 1,775.34		
XR	1255	138	189.76 (29.79-954.62)		
			median = 145.90		
D. pseudoobscura — Pikes Peak					
Chr2	1404	151	164.04 (51.04-758.18)	7	832.80 (482.28-1,087.76)
			median = 148.44		median = 984.34
XL	1399	20	232.03 (187.64-502.21)		
			median = 199.52		
XR	1403	135	188.89 (58.61-964.99)		
			median = 157.46		
D. miranda					
Chr2	1166	148	260.14 (31.91-915.59)	6	890.75 (520.1-1,086.85)
			median = 144.93		median = 998.4
XL	1164	18	230.38 (192.39-394.63)		
			median = 202.90		
XR	1158	114	181.20 (81.28 – 634.60)		
			median = 160.16		
