# Supplementary material for: Recombination Modulates How Selection Affects Linked Sites in Drosophila
Source: PLoS Biol. 2012 Nov 13;10(11):e1001422. doi: 10.1371/journal.pbio.1001422 (PMC3496668; doi:10.1371/journal.pbio.1001422)
Supplement: Table S2 — Uncondensed intervals over which recombination was measured across three recombination maps (D. pseudoobscura–Pikes Peak, D. pseudoobscura–Flagstaff, D. miranda). For “crossovers per individual,” the numbers given are mean/median/mode. “Total Mb covered” is the total distance spanned by the markers used to measure recombination. (PDF) [file pbio.1001422.s015.pdf]

Chromosome 2	*D. pseudoobscura* Pikes Peak	*D. pseudoobscura* Flagstaff	*D. miranda*
Max recombination rate (Kosambi cM/Mb)	30.810	17	23.960
Average recombination rate (Kosambi cM/Mb)	4.054	3.761	5.390
Crossovers per individual	1.053/1/1	1.063/1/1	1.401/1/1
Total Mb covered	30.600	30.119	30.116
% of chromosome	99.4%	97.8%	97.8%
XL			
Max recombination rate (Kosambi cM/Mb)	15.76	6.66	14.25
Average recombination rate (Kosambi cM/Mb)	3.937	4.18	5.435
Crossovers per individual	0.229/0/0	0.496/0/0	0.265/0/0
Total Mb covered	4.641	12.048	4.344
% of chromosome arm	22.8	59.3	21.4
XR			
Max recombination rate (Kosambi cM/Mb)	25.16	21.54	32.33
Average recombination rate (Kosambi cM/Mb)	4.201	3.595	5.5
Crossovers per individual	1.0601/1/1	1.008/1/1	1.540/1/1
Total Mb covered	24.863	26.072	20.656
% of chromosome arm	85.3	89.4	70.8
