# Supplementary material for: Recombination Modulates How Selection Affects Linked Sites in Drosophila
Source: PLoS Biol. 2012 Nov 13;10(11):e1001422. doi: 10.1371/journal.pbio.1001422 (PMC3496668; doi:10.1371/journal.pbio.1001422)
Supplement: Table S3 — Recombination rate for regions of chromosome 2 in Kosambi cM/Mb. The telomere was defined as the end of the chromosome to 2.977 Mb. Centromere was defined as the 27.056 Mb end of chromosome. For the Pikes Peak telomere, the first marker was at 838 bp, whereas for Flagstaff and D. miranda maps, the first markers were at 0.483 Mb and 0.484 Mb, respectively. Using a marker at 0.483 Mb as a start point for Pikes Peak, results in an average telomeric recombination rate of 1.248 Kosambi cM/Mb. (PDF) [file pbio.1001422.s016.pdf]

	D. pseudoobscura Pikes Peak	*D. pseudoobscura* Flagstaff	*D. miranda*
Region			
Telomere	0.738	1.592	1.999
Middle	4.243	4.132	5.615
Centromere	0.479	1.011	1.887
