# Supplementary material for: Recombination Modulates How Selection Affects Linked Sites in Drosophila
Source: PLoS Biol. 2012 Nov 13;10(11):e1001422. doi: 10.1371/journal.pbio.1001422 (PMC3496668; doi:10.1371/journal.pbio.1001422)
Supplement: Table S4 — Chromosome 2 primers used for ultrafine recombination map of Flagstaff 16 backcrossed progeny. All primers amplify loci that differentiate between Flagstaff 16 and Flagstaff 14 by an indel. The location listed is relative to the reference genome of Drosophila pseudoobscura v2.9. Indel, putative indel size in bop; line, line in which putative indel is found. (PDF) [file pbio.1001422.s017.pdf]

**Region 6.1Mb**

| Locus | Location | Size | Indel | Line | Forward primer | Reverse Primer |
|---|---|---|---|---|---|---|
| 6MB_1 | 6,003,085 | 198 | 15 | Flg14 | CCAGGTGCTGCCTATTTTTG | ATACTCGTGGAGCTGGCTGT |
| 6MB_2 | 6,025,001 | 268 | 12 | Flg16 | CGATTGCTGTGTTAGTTTGCTT | GCCGCACAATTCATCATCT |
| 6MB_3 | 6,044,958 | 134 | 33 | Flg14 | GCATGGAATTAATCAACAATCG | AACTAAAGCCCGAGCTGCTG |
| 6MB_4 | 6,062,013 | 264 | 82 | Flg14 | AGTAGGTGTAGAGCCGCACAG | CAGAACACTGCTTTGACATTGA |
| 6MB_5 | 6,083,628 | 212 | 20 | Flg14 | CCAGCGAGAGGAACAAGAGA | CATCAATCAATGCGGAGAGA |
| 6MB_6 | 6,108,295 | 242 | 10 | Flg14 | CGTGCCGTTAAACCAAAATC | CCCACTGAGCACACTCTCAT |

**Region 17.6Mb**

| Locus | Location | Size | Indel | Line | Forward primer | Reverse Primer |
|---|---|---|---|---|---|---|
| 17.6MB_1 | 17,534,400 | 122 | 27 | Flg16 | CTCTGCCATCAATGCCTGTA | CGACTCAAGCAGCTACTTCTCA |
| 17.6MB_2 | 17,555,244 | 207 | 13 | Flg16 | TTGATGATATGATGAAGATTTGTTG | TGCCAAATATGGAGAGTTTTGA |
| 17.6MB_3 | 17,575,208 | 230 | 15 | Flg16 | AAAGAATATTAAAGCTGAAATCTACGA | ATAGGTATATGCAGCAACTTCTGA |
| 17.6MB_4 | 17,594,420 | 207 | 34 | Flg16 | CTGGCATCCATTTTTATTCG | GAGAAAAGTGAAGCAGCGATTT |
| 17.6MB_5 | 17,615,966 | 202 | 32 | Flg16 | TTGCTTCGTTTTATACAATTTTGG | AAAATTGCACCCACGAGACT |
| 17.6MB_6 | 17,638,343 | 250 | 16 | Flg14 | AAGAGAGGGAGGGGTATTTCA | CCAATTCCGAAGCCTAATGA |
| 17.6MB_7 | 17,659,667 | 167 | 17 | Flg14 | ATATCAGTAGGGATACTCATCTTCG | GCAGAAAATCAACATGGATCG |

**Region 21.4Mb**

| Locus | Location | Size | Indel | Line | Forward primer | Reverse Primer |
|---|---|---|---|---|---|---|
| 21.2 MB_2 | 21,437,772 | 180 | 29 | Flg14 | GCTGTTCAGCTACGAAAAACC | GAGTCGGAGCAGAAAACGAA |
| 21.3 MB_3 | 21,465,132 | 269 | 45 | Flg16 | GGTTTAAGATGGTTTTTCCTTTAGAA | GGGCTTTCAACTTTGTTTGG |
| 21.4 MB_4 | 21,488,272 | 147 | 17 | Flg16 | TCGACGGTGGAAACATCTTC | GCACGAAATCCGTAATCGAC |
| 21.5 MB_5 | 21,500,886 | 117 | 12 | Flg16 | GAGGAATTTGTTTACTGCTACCG | AAGGAGAATTAAAACCTTGCACA |
| 21.6 MB_6 | 21,519,392 | 176 | 18 | Flg14 | GGGAAAATTCCCAAGGAAGA | TGAACGATCAATAATTGGCATA |
| 21.8 MB_7 | 21,537,120 | 167 | 36 | Flg16 | CCGTCTGATTTGTTGGTCAT | GCCCCAACATGCAGATAAAA |
