# Supplementary material for: Recombination Modulates How Selection Affects Linked Sites in Drosophila
Source: PLoS Biol. 2012 Nov 13;10(11):e1001422. doi: 10.1371/journal.pbio.1001422 (PMC3496668; doi:10.1371/journal.pbio.1001422)
Supplement: Table S5 — Measures of ultrafine-scale recombination rate and 95% confidence intervals (low cM/Mb and high cM/Mb) for three regions on chromosome 2 constructed from Flagstaff backcrossed progeny described in the text. Values of 0 cM/Mb for the low confidence intervals were used in place of the negative output by the simulations used to calculate the confidence interval. Primers used for ultrafine recombination map are given in Table S4. The marker location listed is relative to the reference genome of Drosophila pseudoobscura v2.9. Interval sizes were confirmed with 76 bp and 9 kb insert mate-paired Illumina reads. Total, total number of individual F2 backcross progeny that were genotyped. (PDF) [file pbio.1001422.s018.pdf]

Region 6.1 Mb

Interval	Marker1	Marker2	Recom.	Total	Mb	cM/Mb	Low cM/Mb	HighcM/Mb
6_1-6_2	6,003,085	6,025,001	22	9667	0.021916	10.38	5.82	19.51
6_2-6_3	6,025,001	6,044,958	9	9665	0.019957	4.67	0	9.68
6_3-6_4	6,044,958	6,062,013	6	9666	0.017055	3.64	0	9.50
6_4-6_5	6,062,013	6,083,628	6	9667	0.021615	2.87	0	7.50
6_5-6_6	6,083,628	6,108,295	15	9666	0.024667	6.29	2.24	14.40

Region 17.6 Mb

Interval	Marker1	Marker2	Recom.	Total	Mb	cM/Mb	Low cM/Mb	HighcM/Mb
17_1-17_2	17,534,400	17,555,244	45	10160	0.020844	21.25	11.65	30.85
17_2-17_3	17,555,244	17,575,208	14	10160	0.019964	6.9	1.89	16.92
17_3-17_4	17,575,208	17,594,420	7	10160	0.019212	3.59	0	8.79
17_4-17_5	17,594,420	17,615,966	9	10160	0.021546	4.11	0	8.75
17_5-17_6	17,615,966	17,638,343	8	10160	0.022377	3.52	0	7.99
17_6-17_7	17,638,343	17,659,667	30	10160	0.021324	13.85	9.16	23.23

Region 21.4 Mb

Interval	Marker1	Marker2	Recom.	Total	Mb	cM/Mb	Low cM/Mb	HighcM/Mb
21_2-21_3	21,437,772	21,465,132	12	10170	0.02736	4.31	0.66	7.97
21_3-21_4	21,465,132	21,488,272	19	10170	0.02314	8.07	3.75	12.4
21_4-21_5	21,488,272	21,500,886	3	10170	0.012614	2.34	0	10.27
21_5-21_6	21,500,886	21,519,392	3	10170	0.018506	1.59	0	7
21_6-21_8	21,519,392	21,537,120	11	10170	0.017728	6.1	0.46	11.74
