# Supplementary material for: Recombination Modulates How Selection Affects Linked Sites in Drosophila
Source: PLoS Biol. 2012 Nov 13;10(11):e1001422. doi: 10.1371/journal.pbio.1001422 (PMC3496668; doi:10.1371/journal.pbio.1001422)
Supplement: Table S6 — Condensed conserved interval information for chromosome 2. (A) Numbers and size of the condensed, conserved intervals between all three maps for chromosome 2. Only chromosome 2 conserved intervals were used for downstream analysis. (B) Average physical differences of marker placement between three maps for the condensed, conserved intervals used in the analysis. All values given are numbers of nucleotides based on the D. pseudoobscura reference genome v2.9. (PDF) [file pbio.1001422.s019.pdf]

	Chr 2	XR
A. Condensed, conserved intervals		
N	27	7
Mean size	305.95 kb	316.89kb
Median size	231.23 kb	295.40kb
Range	104.45 kb – 1,269.70 kb	122.97 - 559.19 kb
B. Differences between marker location across all three maps, conserved intervals		
Range (min, max)	0-5,513 bp	3-2,705 bp
Mean	699 bp	580 bp
Mode	0 bp	116 bp
Median	457 bp	416 bp
