# Supplementary material for: Recombination Modulates How Selection Affects Linked Sites in Drosophila
Source: PLoS Biol. 2012 Nov 13;10(11):e1001422. doi: 10.1371/journal.pbio.1001422 (PMC3496668; doi:10.1371/journal.pbio.1001422)
Supplement: Table S7 — Conserved, condensed intervals. Intervals displayed nonsignificant difference across all three maps when analyzed with a rare events logistic regression and had an Odds Ratio between 0.62 and 1.615 after accounting for the effect of map. Interval windows for each map are given in bp in relation to the reference genome for Drosophila pseudoobscura v2.9. miranda, D. miranda recombination rate; PP, Pikes Peak recombination rate; Flagstaff, Flagstaff recombination rate. The recombination rates given in the table have not been corrected for a global modifier. (PDF) [file pbio.1001422.s020.pdf]

$Start_{mir}$	End_{mir}	*miranda* (cM/Mb)	$Start_{PP}$	End_{PP}	PP (cM/Mb)	$Start_{Flagstaff}$	$End_{Flagstaff}$	Flagstaff (cM/Mb)
1,570,893	2,840,444	0.77	1,570,882	2,840,630	0.50	1,570,899	2,840,604	0.50
4,274,255	5,016,531	9.04	4,275,122	5,015,328	5.50	4,275,122	5,015,853	6.33
5,016,531	5,187,862	5.60	5,015,328	5,189,133	3.67	5,015,853	5,188,165	4.13
5,332,136	5,457,501	7.51	5,331,286	5,457,476	4.48	5,331,286	5,457,476	5.66
5,955,370	6,476,179	10.72	5,954,093	6,476,623	9.94	5,955,452	6,476,292	9.13
8,068,865	8,288,820	7.38	8,068,865	8,287,310	5.34	8,069,015	8,289,379	4.36
8,842,073	8,990,691	4.65	8,842,063	8,990,507	2.61	8,842,146	8,990,478	4.27
8,990,691	9,113,906	5.56	8,990,507	9,113,532	5.65	8,990,478	9,113,429	4.51
9,113,906	9,326,184	3.23	9,113,532	9,325,813	2.00	9,113,429	9,325,684	1.87
11,565,915	11,690,144	5.50	11,567,010	11,689,092	4.06	11,565,072	11,688,998	3.21
11,690,144	11,792,703	10.84	11,689,092	11,795,547	6.74	11,688,998	11,793,452	6.07
12,841,243	13,088,494	8.64	12,838,741	13,087,513	5.35	12,839,350	13,087,513	5.13
13,088,494	13,318,705	3.34	13,087,513	13,318,687	1.60	13,087,513	13,318,738	2.10
14,068,383	14,319,083	2.39	14,068,526	14,318,857	1.41	14,068,547	14,317,723	1.59
14,447,609	14,683,068	3.29	14,447,709	14,682,905	1.80	14,447,629	14,682,905	2.71
14,683,068	14,924,517	7.11	14,682,905	14,924,591	5.27	14,682,905	14,924,507	5.00
14,924,517	15,086,023	20.17	14,924,591	15,088,262	17.74	14,924,507	15,085,361	16.06
15,086,023	15,634,984	7.04	15,088,262	15,635,407	6.60	15,085,361	15,634,802	5.22
16,124,836	16,408,891	7.30	16,126,157	16,407,485	4.27	16,124,907	16,407,237	3.65
16,946,813	17,234,889	2.80	16,941,300	17,236,870	1.44	16,945,779	17,236,917	1.63
17,744,844	17,876,052	5.21	17,743,118	17,876,922	5.32	17,744,561	17,876,465	3.62
18,317,938	18,458,171	4.27	18,317,160	18,459,203	2.49	18,317,447	18,457,739	2.27
18,572,642	18,993,484	3.25	18,574,517	18,994,911	2.06	18,572,663	18,994,021	3.22
19,339,722	20,032,802	7.67	19,340,236	20,032,105	6.14	19,340,226	20,032,029	5.72
20,114,580	20,478,070	3.76	20,114,904	20,478,312	3.57	20,114,724	20,477,555	2.40
22,592,729	22,707,443	4.47	22,592,050	22,708,279	4.87	22,592,041	22,707,899	4.78
25,475,580	25,618,652	11.34	25,474,091	25,619,240	9.26	25,474,138	25,619,483	9.82
