# Supplementary material for: Recombination Modulates How Selection Affects Linked Sites in Drosophila
Source: PLoS Biol. 2012 Nov 13;10(11):e1001422. doi: 10.1371/journal.pbio.1001422 (PMC3496668; doi:10.1371/journal.pbio.1001422)
Supplement: Table S8 — Amount of sequence data obtained for resequenced Drosophila genomes. PE, paired-end. *Total number of reads and base pairs is double the amount listed if “PE” follows run type or if the run type was mate-paired. All data were submitted to the sequence read archive. Accession numbers SRA044960.1, SRA044955.2, and SRA044956.1. (PDF) [file pbio.1001422.s021.pdf]

Strain	Collection locality and date	Species	bp	Reads	Type of Sequencing
Mather 32	Mather, CA; 1997	*D. pseudoobscura*	8,010,565,950	106,807,546	Illumina-75bp
MSH24	Mount St. Helena, CA; 1997	*D. pseudoobscura*	7,699,081,950	102,654,426	Illumina-75bp
MSH9	Mount St. Helena, CA	*D. pseudoobscura*	8,484,305,700	113,124,076	Illumina-75bp
Tree Line	Mather, CA; 1959	*D. pseudoobscura*	6,864,302,025	91,524,027	Illumina-75bp
Pikes Peak 1134	Bosque del Apache National Wildlife Refuge, NM; 2006	*D. pseudoobscura*	7,868,843,700	104,917,916	Illumina-75bp
Pikes Peak 1137	Bosque del Apache National Wildlife Refuge, NM; 2006	*D. pseudoobscura*	9,080,119,350<br>2,412,073,560	121,068,258<br>31,737,810	Illumina-75bp<br>Illumina-76bp mate-paired*
American Fork Canyon 12	American Fork Canyon, UT; 1997	*D. pseudoobscura*	8,296,150,350	110,615,338	Illumina-75bp
Flagstaff18	Flagstaff, AZ; 1997	*D. pseudoobscura*	7,231,357,875	96,418,105	Illumina-75bp
Flagstaff16	Flagstaff, AZ; 1997	*D. pseudoobscura*	3,811,124,520<br>2,470,869,744	108,889,272<br>32,511,444	Illumina-35bp<br>Illumina-76bp mate-paired*
Flagstaff14	Flagstaff, AZ; 1997	*D. pseudoobscura*	3,317,092,975	94,774,085	Illumina-35bp
MV2-25	Genome reference	*D. pseudoobscura*	—	—	—
BogWer		*D. ps. bogotana*	1,280,008,872 PE<br>2,690,495,684 PE	35,555,802 PE<br>35,401,259 PE	Illumina 36 bp PE*<br>Illumina 76 bp PE*
TORO		*D. ps. bogotana*	4,897,006,724 PE	64,434,299 PE	Illumina 76bp PE*

MSH22	Mount St. Helena, CA; 1997	*D. miranda*	767,944,812 PE	10,104,537 PE	Illumina-75bp PE*
SP138		*D. miranda*	1,901,271,480 PE	25,016,730 PE	Illumina-76bp PE*
MAO3.5		*D. miranda*	1,213,232,384 PE	15,963,584 PE	Illumina-76bp PE*
MSH1993	Mount St. Helena, CA; 1993	*D. persimilis*	1,265,090,680 PE<br>2,544,881,775 PE	36,145,448 PE<br>33,931,757 PE	Illumina-35bp PE*<br>Illumina-75bp PE*
MSH3	Genome reference	*D. persimilis*	—	—	—
SCI	Santa Cruz Island, CA SexRatio; 2004	*D. persimilis*	5,224,672,900 PE	104,493,458 PE	Illumina-50bp HiSeq PE*
Lab 3	D. lowei	Lab3Lowei	8,878,213,904<br>1,164,178,184 PE	116,818,604<br>7,659,067 PE	Illumina-75bp<br>Illumina-75bp PE*
