# Supplementary material for: Recombination Modulates How Selection Affects Linked Sites in Drosophila
Source: PLoS Biol. 2012 Nov 13;10(11):e1001422. doi: 10.1371/journal.pbio.1001422 (PMC3496668; doi:10.1371/journal.pbio.1001422)
Supplement: Table S9 — Quasibinomial linear model illustrating the relationship of within-species diversity and between-species divergence at 4-fold degenerate sites of unpreferred codons to various factors for chromosome 2. Neutral mutation rate was the D. persimilis–D. lowei divergence at 4-fold degenerate sites of unpreferred codons. For consistency, interaction terms significant in any of the models were kept in all. Intervals were not condensed across maps and recombination rate was not corrected for a global modifier. (PDF) [file pbio.1001422.s022.pdf]

A) *D. miranda* – Four-fold degenerate sites for unpreferred codons, Chromosome 2:

Response: Within *D. miranda* diversity

	Df	Deviance	Resid.Df	Resid. Dev	F	Pr(>F)
NULL			153	281.46		
Gene Density	1	2.426	152	279.04	1.6283	0.20397
Mutation	1	7.209	151	271.83	4.8394	0.02940 *
**Recombination**	**1**	**33.149**	**150**	**238.68**	**22.2531**	**<0.0001***
GC	1	8.832	149	229.85	5.9286	0.01611*
Gene Density*Mutation	1	5.595	148	224.25	3.7558	0.05457
Gene Density*Recombination	1	2.018	147	222.23	1.3544	0.24642
GC*Mutation	1	4.324	146	217.91	2.9025	0.09058
GC*Recombination	1	1.527	145	216.38	1.0254	0.31294

B) *D. miranda* - Four-fold degenerate sites for unpreferred codons, Chromosome 2:

Response: *D. miranda*-*D. pseudoobscura* divergence

	Df	Deviance	Resid.Df	Resid. Dev	F	Pr(>F)
NULL			153	291.64		
Gene Density	1	0.1275	152	291.52	0.0797	0.778150
Mutation	1	30.0005	151	261.51	18.7390	<0.00001*
**Recombination**	**1**	**0.0879**	**150**	**261.43**	**0.0549**	**0.815105**
GC	1	12.7503	149	248.68	7.9641	0.005442*
Gene Density*Mutation	1	2.6918	148	245.99	1.6813	0.196807
Gene Density*Recombination	1	0.1692	147	245.82	0.1057	0.745599
GC*Mutation	1	3.5827	146	242.23	2.2378	0.136846
GC*Recombination	1	2.3182	145	239.91	1.4480	0.230811

C) Flagstaff recombination map- Four-fold degenerate sites for unpreferred codons, Chromosome 2:

Response: *D. pseudoobscura-D. pseudoobscura* diversity

	Df	Deviance	Resid.Df	Resid. Dev	F	Pr(>F)
NULL			139	365.73		
Gene Density	1	1.203	138	364.53	0.6368	0.42630
Mutation	1	41.299	137	323.23	21.8673	<0.00001*
**Recombination**	**1**	**37.580**	**136**	**285.65**	**19.8980**	<0.00001*
GC	1	17.212	135	268.44	9.1136	0.00305*
Gene Density*Mutation	1	5.129	134	263.31	2.7159	0.10175
Gene Density*Recombination	1	0.767	133	262.54	0.4060	0.52512
GC*Mutation	1	1.163	132	261.38	0.6160	0.43397
GC*Recombination	1	0.334	131	261.05	0.1769	0.67477

D) Flagstaff recombination map- Four-fold degenerate sites for unpreferred codons, Chromosome 2:

Response: *D. miranda-D.pseudoobscura* divergence

	Df	Deviance	Resid.Df	Resid. Dev	F	Pr(>F)
NULL			139	279.92		
Gene Density	1	2.028	138	277.89	1.3105	0.254400
Mutation	1	48.277	137	229.61	31.1908	<0.00001*
**Recombination**	**1**	**0.175**	**136**	**229.44**	**0.1131**	**0.737167**
GC	1	11.796	135	217.64	7.6212	0.006596 *
Gene Density*Mutation	1	6.257	134	211.39	4.0424	0.046423*
Gene Density*Recombination	1	0.205	133	211.18	0.1326	0.716290
GC*Mutation	1	1.228	132	209.95	0.7935	0.374677
GC*Recombination	1	2.246	131	207.71	1.4510	0.230544

E) Pikes Peak recombination map-- Four-fold degenerate sites for unpreferred codons, Chromosome 2:

Response: *D. pseudoobscura-D. pseudoobscura* diversity

	Df	Deviance	Resid.Df	Resid. Dev	F	Pr(>F)
NULL			157	403.80		
Gene Density	1	0.747	156	403.05	0.3962	0.53000
Mutation	1	40.007	155	363.05	21.2219	<0.00001*
**Recombination**	**1**	**42.366**	**154**	**320.68**	**22.4733**	**<0.00001***
GC	1	17.237	153	303.44	9.1433	0.00294*
Gene Density*Mutation	1	1.485	152	301.96	0.7875	0.37629
Gene Density*Recombination	1	7.733	151	294.23	4.1022	0.04461*
GC*Mutation	1	1.479	150	292.75	0.7845	0.37719
GC*Recombination	1	0.154	149	292.59	0.0816	0.77548

F) Pikes Peak recombination map-- Four-fold degenerate sites for unpreferred codons, Chromosome 2:

Response: *D. miranda-D. pseudoobscura* divergence

	Df	Deviance	Resid.Df	Resid. Dev	F	Pr(>F)
NULL			157	315.47		
Gene Density	1	2.392	156	313.08	1.5127	0.220672
Mutation	1	47.810	155	265.27	30.2330	<0.00001*
**Recombination**	**1**	**1.010**	**154**	**264.26**	**0.6389**	**0.425380**
GC	1	12.046	153	252.22	7.6172	0.006508*
Gene Density*Mutation	1	5.578	152	246.64	3.5274	0.062317
Gene Density*Recombination	1	0.362	151	246.28	0.2292	0.632844
GC*Mutation	1	1.433	150	244.84	0.9065	0.342596
GC*Recombination	1	7.712	149	237.13	4.8770	0.028744 *
