# Supplementary material for: Recombination Modulates How Selection Affects Linked Sites in Drosophila
Source: PLoS Biol. 2012 Nov 13;10(11):e1001422. doi: 10.1371/journal.pbio.1001422 (PMC3496668; doi:10.1371/journal.pbio.1001422)
Supplement: Table S10 — Quasibinomial linear model illustrating the relationship of within-species diversity and between-species divergence at 4-fold degenerate sites of unpreferred codons to various factors for the XR chromosome arm. Neutral mutation rate was the D. persimilis–D. lowei divergence at 4-fold degenerate sites for unpreferred codons sites. For consistency, interaction terms significant in any of the models were kept in all. Intervals were not condensed across maps, and recombination rate was not corrected for a global modifier. (PDF) [file pbio.1001422.s023.pdf]

A) *D. miranda* - Four-fold degenerate sites for unpreferred codons, XR chromosome arm:

Response: Within *D. miranda* diversity

	Df	Deviance	Resid.Df	Resid. Dev	F	Pr(>F)
NULL			113	189.31		
Gene Density	1	19.3165	112	169.99	14.7325	0.000212*
Mutation	1	4.0318	111	165.96	3.0750	0.082422
**Recombination**	**1**	**3.6666**	**110**	**162.29**	**2.7965**	**0.097450**
GC	1	12.0835	109	150.21	9.2159	0.003026*
Gene Density*Mutation	1	1.7380	108	148.47	1.3256	0.252209
Gene Density*Recombination	1	2.2921	107	146.18	1.7482	0.188979
GC*Mutation	1	4.1785	106	142.00	3.1869	0.077120
GC*Recombination	1	0.2356	105	141.77	0.1797	0.672503

B) *D. miranda* - Four-fold degenerate sites for unpreferred codons, XR chromosome arm:

Response: *D. pseudoobscura- D. miranda* divergence

	Df	Deviance	Resid.Df	Resid. Dev	F	Pr(>F)
NULL			113	150.03		
Gene Density	1	0.4358	112	149.60	0.4091	0.52382
Mutation	1	25.7548	111	123.84	24.1743	<0.00001*
**Recombination**	**1**	**2.2430**	**110**	**121.60**	**2.1054**	**0.14976**
GC	1	6.2611	109	115.34	5.8769	0.01705 *
Gene Density*Mutation	1	0.0689	108	115.27	0.0646	0.79979
Gene Density*Recombination	1	1.8244	107	113.44	1.7124	0.19353
GC*Mutation	1	1.4378	106	112.01	1.3496	0.24798
GC*Recombination	1	0.1440	105	111.86	0.1352	0.71385

C) *D. pseudoobscura* – Flagstaff- Four-fold degenerate sites for unpreferred codons, XR chromosome arm:

Response: Within *D. pseudoobscura* diversity

	Df	Deviance	Resid.Df	Resid. Dev	F	Pr(>F)
NULL			136	340.12		
Gene Density	1	0.9072	135	339.21	0.4275	0.514404
Mutation	1	13.1025	134	326.11	6.1740	0.014255 *
**Recombination**	**1**	**16.3881**	**133**	**309.72**	**7.7222**	**0.006276 ***
GC	1	7.8689	132	301.85	3.7079	0.056372
Gene Density*Mutation	1	6.7494	131	295.10	3.1804	0.076898
Gene Density*Recombination	1	2.1753	130	292.93	1.0250	0.313240
GC*Mutation	1	1.9675	129	290.96	0.9271	0.337426
GC*Recombination	1	0.1473	128	290.81	0.0694	0.792639

D) *D. pseudoobscura* – Flagstaff- Four-fold degenerate sites for unpreferred codons, XR chromosome arm:

Response: *D. pseudoobscura*- *D. miranda* divergence

	Df	Deviance	Resid.Df	Resid. Dev	F	Pr(>F)
NULL			136	191.95		
Gene Density	1	0.061	135	191.89	0.0583	0.809536
Mutation	1	37.210	134	154.68	35.5459	<0.00001*
**Recombination**	**1**	**0.219**	**133**	**154.46**	**0.2096**	**0.647832**
GC	1	10.574	132	143.89	10.1008	0.001858 *
Gene Density*Mutation	1	0.022	131	143.86	0.0207	0.885836
Gene Density*Recombination	1	0.171	130	143.69	0.1632	0.686941
GC*Mutation	1	4.749	129	138.94	4.5362	0.035099 *
GC*Recombination	1	0.014	128	138.93	0.0130	0.909472

E) *D. pseudoobscura* – Pikes Peak- Four-fold degenerate sites for unpreferred codons, XR chromosome arm:

Response: Within *D. pseudoobscura* diversity

	Df	Deviance	Resid.Df	Resid. Dev	F	Pr(>F)
NULL			130	348.84		
Gene Density	1	8.867	129	339.98	4.4927	0.036066 *
Mutation	1	23.251	128	316.72	11.7803	0.000818*
**Recombination**	**1**	**16.148**	**127**	**300.58**	**8.1813**	**0.004981***
GC	1	32.477	126	268.10	16.4545	<0.00001*
Gene Density*Mutation	1	19.330	125	248.77	9.7939	0.002191*
Gene Density*Recombination	1	2.887	124	245.88	1.4626	0.228853
GC*Mutation	1	0.916	123	244.97	0.4639	0.497078
GC*Recombination	1	0.000	122	244.97	0.0001	0.993139

F) *D. pseudoobscura* – Pikes Peak- Four-fold degenerate sites for unpreferred codons, XR chromosome arm:

Response: *D. pseudoobscura*- *D. miranda* divergence

	Df	Deviance	Resid.Df	Resid. Dev	F	Pr(>F)
NULL			130	186.13		
Gene Density	1	0.017	129	186.11	0.0172	0.89577
Mutation	1	40.047	128	146.06	40.2343	<0.00001*
**Recombination**	**1**	**0.094**	**127**	**145.97**	**0.0944**	**0.75922**
GC	1	16.499	126	129.47	16.5766	<0.00001*
Gene Density*Mutation	1	3.239	125	126.23	3.2540	0.07372
Gene Density*Recombination	1	0.130	124	126.10	0.1308	0.71823
GC*Mutation	1	2.508	123	123.59	2.5201	0.11499
GC*Recombination	1	0.086	122	123.51	0.0862	0.76954
