# Supplementary material for: Recombination Modulates How Selection Affects Linked Sites in Drosophila
Source: PLoS Biol. 2012 Nov 13;10(11):e1001422. doi: 10.1371/journal.pbio.1001422 (PMC3496668; doi:10.1371/journal.pbio.1001422)
Supplement: Table S11 — Quasibinomial linear model illustrating the relationship of within-species diversity and between-species divergence at 4-fold degenerate sites of unpreferred codons to various factors for the XL chromosome arm. Neutral mutation rate was the D. persimilis–D. lowei divergence at 4-fold degenerate sites of unpreferred codons. For consistency, interaction terms significant in any of the models were kept in all. Intervals were not condensed across maps, and recombination rate was not corrected for a global modifier. Only D. miranda and D. pseudoobscura–Pikes Peak are given because there were too few intervals for the D. pseudoobscura–Flagstaff map to perform the analysis. (PDF) [file pbio.1001422.s024.pdf]

A) *D. miranda* - Four-fold degenerate sites for unpreferred codons, XL chromosome arm:

Response: Within *D. miranda* diversity

	Df	Deviance	Resid.Df	Resid. Dev	F	Pr(>F)
NULL			17	25.3338		
Gene Density	1	0.7578	16	24.5760	0.9615	0.35242
Mutation	1	1.0581	15	23.5179	1.3425	0.27641
**Recombination**	**1**	**2.0402**	**14**	**21.4778**	**2.5886**	**0.14209**
GC	1	0.0573	13	21.4205	0.0727	0.79354
Gene Density*Mutation	1	1.3210	12	20.0994	1.6762	0.22766
Gene Density*Recombination	1	7.2513	11	12.8481	9.2008	0.01417 *
GC*Mutation	1	0.6616	10	12.1865	0.8394	0.38345
GC*Recombination	1	5.8711	9	6.3154	7.4495	0.02325 *

B) *D. miranda* - Four-fold degenerate sites for unpreferred codons, XL chromosome arm:

Response: *D. pseudoobscura*- *D. miranda* divergence

	Df	Deviance	Resid.Df	Resid. Dev	F	Pr(>F)
NULL			17	41.125		
Gene Density	1	12.6362	16	28.489	6.7677	0.02867 *
Mutation	1	0.4304	15	28.058	0.2305	0.64258
**Recombination**	**1**	**1.4995**	**14**	**26.559**	**0.8031**	**0.39351**
GC	1	10.9844	13	15.574	5.8831	0.03826 *
Gene Density*Mutation	1	0.7721	12	14.802	0.4135	0.53623
Gene Density*Recombination	1	0.0077	11	14.795	0.0041	0.95034
GC*Mutation	1	0.0018	10	14.793	0.0010	0.97605
GC*Recombination	1	0.3844	9	14.408	0.2059	0.66074

C) *D. pseudoobscura* – Pikes Peak- Four-fold degenerate sites for unpreferred codons, XL chromosome arm:

Response: Within *D. pseudoobscura* diversity

	Df	Deviance	Resid.Df	Resid. Dev	F	Pr(>F)
NULL			18	42.276		
Gene Density	1	8.0291	17	34.247	10.7668	0.0082711*
Mutation	1	0.4097	16	33.837	0.5494	0.4755915
**Recombination**	**1**	**19.2485**	**15**	**14.588**	**25.8116**	**0.0004774***
GC	1	1.5211	14	13.067	2.0397	0.1837143
Gene Density*Mutation	1	3.1366	13	9.931	4.2062	0.0674077
Gene Density*Recombination	1	1.5680	12	8.363	2.1026	0.1776799
GC*Mutation	1	0.6674	11	7.695	0.8949	0.3664408
GC*Recombination	1	0.2394	10	7.456	0.3210	0.5835086

D) *D. pseudoobscura* – Pikes Peak- Four-fold degenerate sites for unpreferred codons, XL chromosome arm:

Response: *D. pseudoobscura*- *D. miranda* divergence

	Df	Deviance	Resid.Df	Resid. Dev	F	Pr(>F)
NULL			18	37.157		
Gene Density	1	8.1702	17	28.987	5.8387	0.03629 *
Mutation	1	0.0643	16	28.922	0.0459	0.83458
**Recombination**	**1**	**2.4114**	**15**	**26.511**	**1.7233**	**0.21860**
GC	1	11.0000	14	15.511	7.8609	0.01867 *
Gene Density*Mutation	1	0.6197	13	14.891	0.4429	0.52079
Gene Density*Recombination	1	0.0485	12	14.843	0.0347	0.85605
GC*Mutation	1	0.1080	11	14.735	0.0772	0.78677
GC*Recombination	1	0.6295	10	14.105	0.4499	0.51759
