# Supplementary material for: Recombination Modulates How Selection Affects Linked Sites in Drosophila
Source: PLoS Biol. 2012 Nov 13;10(11):e1001422. doi: 10.1371/journal.pbio.1001422 (PMC3496668; doi:10.1371/journal.pbio.1001422)
Supplement: Table S12 — Mean and standard deviation for each factor in the models presented in Table 5 and Table 6. (PDF) [file pbio.1001422.s025.pdf]

	Table 5: nonsynonymous substitutions		Table 6: synonymous substitutions	
	**mean**	**stdev**	**mean**	**stdev**
Eligible bases	119.0559	26.87643	118.3707	26.21697
GC content	0.5243242	0.05096841	0.5261032	0.04956667
Neutral mutation rate	0.07357141	0.02968777	0.06502924	0.02874945
Proportion coding	0.79597	0.1573495	0.8003886	0.155288
Proportion nonsynonymous	0.003731609	0.006091581	0.003205379	0.005270208
Absolute distance	26741.45	18037.03	26299.4	18043.82
Recombination rate	3.285556	2.499529	3.057299	2.547031
