# Supplementary material for: Recombination Modulates How Selection Affects Linked Sites in Drosophila
Source: PLoS Biol. 2012 Nov 13;10(11):e1001422. doi: 10.1371/journal.pbio.1001422 (PMC3496668; doi:10.1371/journal.pbio.1001422)
Supplement: Text S1 — Supporting information and methods. (DOCX) [file pbio.1001422.s026.docx]

**Supplementary Materials for**

**“**Recombination modulates how selection affects linked sites in *Drosophila***”**

Suzanne E. McGaugh, Caitlin Smukowski, Brenda Manzano-Winkler, Laurence Loewe, Steve Goldstein, Tiffany Himmel, Mohamed A. F. Noor

**Supplementary Materials & Methods**

*Study system*

The *Drosophila pseudoobscura* group inhabits western North America and contains six species [[1](#_ENREF_1)] (Figure S2). *Drosophila pseudoobscura* and *D. persimilis* diverged from one another 500,000 years ago [[2](#_ENREF_2)] and both diverged from *D. miranda* ~2 mya [[3](#_ENREF_3)]. The most closely related available outgroup of these three species is *D. lowei* which diverged from *D. pseudoobscura* approximately 5-11mya [[1](#_ENREF_1)]. One subspecies of *Drosophila pseudoobscura*, *Drosophila pseudoobscura bogotana*, has been isolated in Colombia for ~ 200,000 years ago [[2](#_ENREF_2),[4](#_ENREF_4)]. In this work, we generated linkage maps for chromosome 2 and parts of the X chromosome for *D. pseudoobscura* and *D. miranda*.

Members of the *pseudoobscura* group have six chromosome arms (three telocentric autosomes [chromosomes 2-4], one “dot” autosome (chromosome 5), and a metacentric X chromosome), comprising their ~160Mb genome [[5](#_ENREF_5)]. Chromosome 3 segregates for more than 30 different inversion arrangements within *D. pseudoobscura* [[6](#_ENREF_6)] and the dot chromosome does not recombine; therefore both were excluded in our study. *Drosophila pseudoobscura* average crossover rate is about 7 cM/Mb in females (over twice that of *D. melanogaster*) [[7](#_ENREF_7)].

*Fine-scale crossover maps: Crosses and technical details*

The first *D. pseudoobscura* map was generated using two inbred lines of flies that are homozygous for the Arrowhead arrangement on chromosome 3. Briefly, *D. pseudoobscura* females from the Flagstaff 16 line (collected from Flagstaff, AZ in 1997) were crossed with males of Flagstaff 14 (Flagstaff, AZ, 1997). F1 virgin females were backcrossed to Flagstaff 16 males. The second *D. pseudoobscura* map was generated using two inbred lines of flies that are homozygous for the Pikes Peak arrangement on chromosome 3. *Drosophila pseudoobscura* females from the Pikes Peak 1137 line (collected from Bosque del Apache National Wildlife Refuge, San Antonio, NM in 2006) were crossed with males of the Pikes Peak 1134 line (Bosque del Apache National Wildlife Refuge, San Antonio, NM, 2006). F1 virgin females were backcrossed to Pikes Peak 1137 males. Likewise, in *D. miranda*, inbred MSH22 (Mt. St. Helena, CA) females were crossed with inbred SP138 (Spray, OR) males. F1 virgin females were backcrossed to MSH22 males.

Progeny from these backcrosses were stored in 96-well plates, frozen at -20ºC, and transferred to North Carolina State University Genomic Sciences Laboratory for DNA extraction via Invitrogen ChargeSwitch® gDNA Micro Tissue Kit (cat# CS11203 Carlsbad, CA, USA).

*Fine-scale crossover maps: SNP development for genotyping arrays*

Genomic DNA Illumina reads were obtained for 15-20 virgin female inbred flies from University of North Carolina-Chapel Hill High Throughput Sequencing Facility for all lines described in the crosses above (see Table S7). SNPs were identified from this Illumina data for genotyping using the Illumina BeadArray platform [[8](#_ENREF_8)] (Illumina, San Diego, California, United States).

For the Flagstaff cross, Ilumina single-end 35bp reads were aligned to the reference *D. pseudoobscura* genome v2.6 via Bowtie 0.10.1 [[9](#_ENREF_9)]. The options “-a --best –strata” were employed to filter the output to contain the least number of mismatches to the reference, and only two mismatches between a single read and the reference were allowed. A series of custom python scripts identified bases that fit the following criteria:

1) at least 50% of the Illumina reads supported the SNP relative to the reference;

2) the average quality score was less than or equal to a 1% error rate;

3) line 1and line 2 did not have a shared polymorphism relative to the reference; and

4) no other high-quality SNPs or ambiguous bases were found in the 50bp flanking the SNP for

either line, as this would interfere with the downstream genotyping assay.

SNPs and their flanking regions were scored by Illumina’s Assay Design Tool to determine their suitability for genotyping with Illumina’s BeadArray platform. A SNP was not used if it had a final score less than 0.7 or designability rank less than 1. We selected a final set of 384 SNPs and confirmed a small subset (< 10) of these SNPs with Sanger sequencing.

For the Pikes Peak and *D. miranda* recombination maps, Illumina single-end 75bp reads and 76bp paired end reads, respectively, (see Table S7) were aligned to the *D. pseudoobscura* reference genome v2.9 using bwa-0.5.5 (default alignment settings were used except the maximum number of gap extensions was set to 4 [[10](#_ENREF_10)]). Using bwa, as opposed to Bowtie, provided the advantage of identifying indels and excluding them from SNP development. Consensus assemblies for each resequenced line were generated using the bwa alignments and Samtools 0.1.6 pileup [[11](#_ENREF_11)]. Default pileup settings were used except the theta parameter (error dependency coefficient) in the maq consensus calling model was set to 0.9, and the number of haplotypes in the sample was set to one because each resequenced line was inbred for > 5 generations prior to sequencing.

For the Pikes Peak and *D. miranda* recombination maps, consensus sequences for each of the inbred lines were filtered with custom python scripts to ensure:

1) line 1 and line 2 did not have a shared polymorphism relative to the reference,

2) neither line 1 or line 2 had an indel denoted as the focal SNP nucleotide and

3) no other high-quality SNPs, ambiguous bases, or indels were found in the 50bp flanking the SNP as this would interfere with the genotyping assay.

No additional filtering on quality or depth of coverage was used on the pileup consensus assembly when selecting these SNPs. SNPs and their flanking regions were scored by Illumina’ s Assay Design Tool, and any SNP with a final score less that 0.7 or designability rank less than 1 was not used. We selected a final set of 384 SNPs by visualizing the best candidates in Integrative Genomics Viewer v1.4.04 [[12](#_ENREF_12)]. Sanger sequencing was used to confirm a small subset (< 10) of SNPs. Priority was given to markers that were located near one another across all three recombination maps to ensure that we measured recombination over similar intervals between maps.

*Fine-scale recombination maps: Condensing intervals*

Intervals between all three fine-scale maps were condensed so that the markers for each map were at the same approximate genomic position. Ninety-seven intervals remained for chromosome 2 and 44 intervals for XR (see Table S6 for number of individuals, size, and range of the condensed, conserved intervals). Recombination was estimated as detailed in the Materials and Methods section, using the number of crossovers spanning the newly defined physical interval, and if the number of individuals surveyed differed across the intervals condensed, the least number of individuals was used. Details of how conserved and divergent intervals were defined are available in the Materials and Methods.

Intervals were also condensed between maps in a pairwise fashion (Flagstaff-Pikes Peak, Flagstaff-*D. miranda*, Pikes Peak-*D. miranda*). Because they only required markers to be identical across two maps, these pairwise comparisons were able to compare recombination rates between maps on a finer scale.

*Fine-scale recombination maps: Computational methods for diversity and divergence measures*

To generate diversity and divergence correlates of recombination rate, we utilized resequenced genomes from lines of the *pseudoobscura* clade that were inbred as single-pair, full-sibling matings for at least five generations, though most resequenced lines were inbred in this manner for 15 generations. Those lines that were inbred for only five generations had undergone some inbreeding prior to this, because they were made homozygous for particular chromosome three inversions prior to inbreeding for this study. We also included the reference genomes of *D. pseudoobscura* v.2.9 and v.1.3 *D. persimilis* in our calculations. Illumina reads from resequenced genomes of *Drosophila pseudoobscura* (N = 10), *D. ps. bogotana* (N = 2), *D. persimilis* (N = 2), *D. miranda* (N = 3), and one *D. lowei* (N = 1) (Table S7) were each aligned separately to the reference *D. pseudoobscura* genome v2.9 with bwa, and a pileup consensus output was generated with Samtools (as described above). Prior to measuring pairwise diversity and divergence within each recombination interval, pileup consensus alignments were filtered for each line in the following ways:

1) read depth must be four or larger,

2) consensus quality must be at least 31,

3) indels were taken as ‘real’ if they were supported by at least 70% of reads,

4) if indels are supported by at least 70% of reads, 5 bases on either side of the indel bases and the indel bases were excluded from the analysis.

Nucleotides that went through these filtering requirements were considered ‘eligible bases’ for downstream analyses. No additional filtering on quality or depth of coverage was used on the pileup consensus assembly when selecting SNPs. Intergenic regions, exons, and introns from the reference genome v1.3 for *D. persimilis* were aligned to the *D. pseudoobscura* reference using bwa in the same manner described above.

FlyBase annotations of the *Drosophila pseudoobscura* genome v.2.9 were used to demarcate four-fold degenerate coding positions for unpreferred codons. Unpreferred codons defined as in [[13](#_ENREF_13)]. Bases annotated as an exon on one strand and as intronic or intergenic nucleotides on the other strand were treated only as exonic nucleotides. Genes whose coding region on one strand overlapped with the coding region of another gene on the other strand were excluded because it is unlikely that the site would be a four-fold degenerate site of unpreferred codons in both sequences. To calculate pairwise sequence diversity between two lines, bases which were polymorphic (SNP) and eligible bases which were not polymorphic between the two lines (non-SNP) were counted for each recombination interval. The analysis using diversity or divergence presented in the paper mainly deals with fourfold degenerate sites of unpreferred codons. Bases were only included if at least one representative from each species or subspecies contained an eligible base at the site being compared. We calculated π by making all pairwise comparisons for each interval (e.g. for *D. pseudoobscura* diversity all pairwise comparisons between the 11 lines were made). The number of SNP bases was averaged across each recombination interval for all pairwise comparisons. Likewise, the number of non-SNP bases was averaged across each recombination interval for all pairwise comparisons. Since we aligned the reference genome contigs of *D. persimilis* to *D. pseudoobscura* instead of Illumina sequenced reads for MSH3 (the reference genome strain), we required that one other *D. persimilis* line have eligible bases in order for the MSH3 bases to be included.

*Fine-scale recombination maps: Recombination, diversity and divergence*

Neutral divergence can be used as a proxy for mutation rate [[14](#_ENREF_14),[15](#_ENREF_15)]. We can account for the effect of the heterogeneity in mutation rate across the genome on diversity by including neutral divergence measures into a logistic regression (GLM with logit link function) with neutral diversity as the response variable (*sensu* Wilson and Hardy, 2002; Warton and Hui, 2010) (e.g. R code: cbind(# of SNP bases, # of non-SNP bases) ). We measured the average level of divergence between *D. persimilis* and *D. lowei* at four fold degenerate sites of unpreferred codons. We focused on divergence between these species because they are close enough to *D. pseudoobscura* to share similar variation in mutation rates along the genome; however, the divergence between *D. persimilis* and *D. pseudoobscura* lineage is limited, which means these divergence estimates are somewhat confounded with diversity measurements [[16](#_ENREF_16)] . This divergence measure was used to account for neutral mutation rate variation across the genome and is separate from the divergence measures used between *D. miranda* and *D. pseudoobscura*, which were used for testing for a relationship between recombination and divergence. Including this divergence measure in our models, since it is likely slightly confounded with diversity of *D. pseudoobscura*, produces a more conservative result.

*Ultrafine crossover maps: Marker development and recombination map construction*

Ultrafine scale crossover maps were generated for three regions along chromosome 2, placing a marker every ~20kb. In total, 20 indel markers within three regions across chromosome 2, were designed manually by viewing sorted bam files generated from bwa alignments in Integrative Genomics Viewer v1.4.04 [[12](#_ENREF_12)]. Their coordinates on chromosome 2 are: 6.003 Mb- 6.108 Mb (6 markers, 5 intervals, average interval 20.280 Kbp), 17.534 MB - 17.660 MB (7 markers, 6 intervals, average interval 20.878 Kbp), 21.438 Mb -21.537 Mb (6 markers, 5 intervals, average interval 19.870 Kbp) (see Table S4, S5). For brevity, these regions will be referred to as 6Mb, 17Mb, and 21Mb. Distance between markers was confirmed by aligning 76bp, 9kb mate-paired Illumina reads to the reference genome with bwa and ensuring that insert sizes were as expected for multiple read pairs.

Using Flagstaff 16 and Flagstaff 14, we followed the same backcross scheme described in *Fine-scale crossover maps: Crosses and technical details* section. Over 10,000 progeny from this backcross were stored in 96-well plates and frozen at -20°C. Flies were DNA prepped using 63.5ul squish buffer (10mM Tris-HCl (pH 8.2), 1mM EDTA, 25mM NaCl) + 1.3 proteinase K [[17](#_ENREF_17)], a Zirconium bead was placed in each well, and packaging tape was sealed over the plate for 7 minutes at 23°C. Plates were then shaken using a Qiagen TissueLyser II for 45 seconds.

The PCR recipe generally consisted of 0.5 uM of forward primer +M13 tag, 0.5 uM of reverse primer, 0.1 uM of 700IRD or 800IRD-labeled M13 tag, 1.5 mM MgCl2, 1 X buffer, 0.2 mM dNTPs, and 1U of taq in a 10 uL reaction volume. Generally, the PCR program consisted of an initial denaturing step of 94ºC for 60 sec, three touch-down cycles of 94ºC- 58ºC- 72ºC for 30 sec each, followed by 31 main cycles of 94ºC- 56ºC- 72ºC for 30 sec each. Products were visualized on a polyacrylamide gel using LICOR 4300.

In total, 9,657 individuals were scored for the 6Mb region, 10,160 individuals were scored for the 17Mb region, and 10,170 were scored for the 21Mb region. 95 % confidence intervals for recombination rate for each recombination interval were calculated by permutation [[18](#_ENREF_18),[19](#_ENREF_19)] and are given in the Supplementary Information. For All 9,657 individuals were genotyped for the six markers of the 6Mb region (Table S4, S5). No double recombinants were found across the 6Mb region, therefore, for the 17Mb and 21Mb region the markers on the edge of each region were genotyped (e.g. 17_1, 17_7; Table S4, S5) and individuals identified as being recombinants were further genotyped for internal markers (e.g. 17_2 - 17_6; Table S4, S5) to identify where in the region the recombination event took place.

*Recombination and selection*

Recombination and nonsynonymous substitutions

To test for a correlation between recombination rate and protein evolution, synonymous and nonsynonymous substitutions, specific to the *D. pseudoobscura* + *D. persimilis* lineage, were calculated for each gene using PAML v4.4d [[20](#_ENREF_20)]. We estimated the ancestral sequences and identifying synonymous and nonsynonymous substitutions along the branch to *D. pseudoobscura* + *D. persimilis*. This method is very similar to that employed by [[21](#_ENREF_21)]. All of the resequenced genomic and reference genomic data described above (one *D. lowei*, three *D. miranda*, three *D. persimilis*, two *D. pseudoobscura bogotana* and 11 *D. pseudoobscura* genomes, filtered for quality as described above) was used. We used a tree rooted with *D. lowei* and considered the branches leading to (*D. persimilis* (*D. pseudoobscura*, *D. pseudoobscura bogotana*)) to be the foreground branches. We included *D. persimilis* a part of the foreground branch because relatively extensive interbreeding occurs between *D. pseudoobscura* and *D. persimilis* across much of the genome, aside from a few inverted regions [[22-24](#_ENREF_22)]. We calculate the ancestral synonymous and nonsynonymous substitutions using a modified model A from Nielsen and Yang (2002) , with the following restrictions: ω (d_N_/d_S_) was not constrained to be equal on all branches of the tree, ω was fixed at 1 along the foreground branch, codon frequencies were estimated from the nucleotide frequencies observed in the data (F3X4 method), transition-transversion ratio was estimated for each gene, a single α for all sites was assumed, and branch lengths were estimated with maximum likelihood from starting values. Genes with premature stop codons in multiple *D. pseudoobscura* lines or genes that did not have sufficient coverage in at least eight *D. pseudoobscura* genomes and one genome from each: *D. lowei*, *D. pseudoobscura bogotana*, *D. persimilis,* and *D. miranda* were excluded from this analysis.

**References**

1. Beckenbach AT, Wei YW, Liu H (1993) Relationships in the Drosophila obscura species group, inferred from mitochondrial cytochrome oxidase II sequences. Molecular Biology and Evolution 10: 619-634.

2. Wang RL, Wakeley J, Hey J (1997) Gene flow and natural selection in the origin of Drosophila pseudoobscura and close relatives. Genetics 147: 1091-1091.

3. Barrio E, Latorre A, Moya A, Ayala FJ (1992) Phylogenetic reconstruction of the Drosophila obscura group, on the basis of mitochondrial DNA. Molecular biology and evolution 9: 621-621.

4. Schaeffer SW, Miller EL (1991) Nucleotide sequence analysis of Adh genes estimates the time of geographic isolation of the Bogota population of Drosophila pseudoobscura. Proceedings of the National Academy of Sciences 88: 6097-6097.

5. Gregory TR, Johnston JS (2008) Genome size diversity in the family Drosophilidae. Heredity 101: 228-238.

6. Dobzhansky T, Powell JR (1975) Drosophila pseudoobscura and its American relatives, Drosophila persimilis and Drosophila miranda. Handbook of Genetics 3: 537-587.

7. Ortiz-Barrientos D, Chang AS, Noor MAF (2006) A recombinational portrait of the *Drosophila pseudoobscura* genome. Genetics Research 87: 23-31.

8. Fan JB, Oliphant A, Shen R, Kermani BG, Garcia F, et al. Highly parallel SNP genotyping; 2003. pp. 69-69.

9. Langmead B, Trapnell C, Pop M, Salzberg SL (2009) Ultrafast and memory-efficient alignment of short DNA sequences to the human genome. Genome Biol 10: R25-R25.

10. Li H, Durbin R (2009) Fast and accurate short read alignment with Burrows–Wheeler transform. Bioinformatics 25: 1754-1754.

11. Li H, Handsaker B, Wysoker A, Fennell T, Ruan J, et al. (2009) The Sequence alignment/map (SAM) format and SAMtools. Bioinformatics.

12. Robinson JT, Thorvaldsdóttir H, Winckler W, Guttman M, Lander ES, et al. (2011) Integrative genomics viewer. Nature Biotechnology 29: 24-26.

13. Vicario S, Moriyama EN, Powell J (2007) Codon usage in twelve species of *Drosophila*. BMC Evolutionary Biology 7: 226.

14. Kimura M (1983) The neutral theory of molecular evolution: Cambridge University Press.

15. Birky CW, Walsh JB (1988) Effects of linkage on rates of molecular evolution. Proceedings of the National Academy of Sciences 85: 6414-6418.

16. Sattath S, Elyashiv E, Kolodny O, Rinott Y, Sella G (2011) Pervasive Adaptive Protein Evolution Apparent in Diversity Patterns around Amino Acid Substitutions in Drosophila simulans. PLoS Genetics 7: e1001302-e1001302.

17. Gloor GB, Engels WR (1992) Single fly DNA preps for PCR. Drosophila Information Service 71: 148-149.

18. Cirulli ET, Kliman RM, Noor MAF (2007) Fine-scale crossover rate heterogeneity in *Drosophila pseudoobscura*. Journal of Molecular Evolution 64: 129-135.

19. Stevison LS, Noor MAF (2010) Genetic and evolutionary correlates of fine-scale recombination rate variation in *Drosophila persimilis*. Journal of Molecular Evolution 71: 332-345.

20. Yang Z (2007) PAML 4: a program package for phylogenetic analysis by maximum likelihood. Molecular Biology and Evolution 24: 1586-1591.

21. Sattath S, Elyashiv E, Kolodny O, Rinott Y, Sella G (2011) Pervasive adaptive protein evolution apparent in diversity patterns around amino acid substitutions in *Drosophila simulans*. PLoS Genetics 7: e1001302.

22. Machado CA, Haselkorn TS, Noor MAF (2007) Evaluation of the genomic extent of effects of fixed inversion differences on intraspecific variation and interspecific gene flow in Drosophila pseudoobscura and D. persimilis. Genetics 175: 1289-1289.

23. Noor MAF, Garfield DA, Schaeffer SW, Machado CA (2007) Divergence between the Drosophila pseudoobscura and D. persimilis genome sequences in relation to chromosomal inversions. Genetics 177: 1417-1417.

24. McGaugh SE, Noor MAF (2012) Genomic impacts of chromosomal inversions in parapatric Drosophila species Phil Trans R Soc B 367: 422-429.
